# Brain Age Estimation on T2‐FLAIR Scans for Application to Multiple Sclerosis

**DOI:** 10.1002/hbm.70425

**Published:** 2026-03-19

**Authors:** Jordan Colman, Giuseppe Pontillo, Olivia Goodkin, Michael A. Foster, Nima Mahmoudi, Mike P. Wattjes, Arturo Brunetti, Gabriel Gonzalez‐Escamilla, Sergiu Groppa, Einar August Høgestøl, Lars T. Westlye, Silvia Messina, Jacqueline Palace, Rosa Cortese, Nicola De Stefano, Àlex Rovira, Jaume Sastre‐Garriga, Stefan Ropele, Christian Enzinger, Maria A. Rocca, Massimo Filippi, Barbara Bellenberg, Carsten Lukas, Massimiliano Calabrese, Marco Castellaro, Tomas Uher, Manuela Vaneckova, Ahmed Toosy, Olga Ciccarelli, Tarek Yousry, Ferran Prados, Frederik Barkhof, James H. Cole, MAGNIMS Study Group, Frederik Barkhof, Nicola de Stefano, Jaume Sastre‐Garriga, Olga Ciccarelli, Christian Enzinger, Massimo Filippi, Claudio Gasperini, Cristina Granziera, Gabriele Deluca, Menno M. Schoonheim, Alex Rovira, Maria A. Rocca, Ahmed Toosy

**Affiliations:** ^1^ UCL Hawkes Institute, University College London London UK; ^2^ Department of Neuroradiology King's College Hospital London UK; ^3^ Queen Square Multiple Sclerosis Centre, Department of Neuroinflammation UCL Queen Square Institute of Neurology London UK; ^4^ MS Center Amsterdam, Radiology and Nuclear Medicine, Vrije Universiteit Amsterdam, Amsterdam Neuroscience, Amsterdam UMC Location VUmc Amsterdam the Netherlands; ^5^ Department of Advanced Biomedical Sciences University of Naples “Federico II” Naples Italy; ^6^ Bayer Plc Reading UK; ^7^ Department of Neuroradiology Charité—Universitätsmedizin Berlin, Corporate Member of Freie Universität Berlin, Humboldt‐Universität Zu Berlin Berlin Germany; ^8^ Movement Disorders, Neurostimulation and Neuroimaging University Medicine Mainz Mainz Germany; ^9^ Department of Neurology Oslo University Hospital Oslo Norway; ^10^ Department of Psychology University of Oslo Oslo Norway; ^11^ Institute of Clinical Medicine, University of Oslo Oslo Norway; ^12^ Nuffield Department of Clinical Neurosciences Medical Sciences Division, University of Oxford Oxford UK; ^13^ Department of Medicine Surgery and Neuroscience, University of Siena Siena Italy; ^14^ Section of Neuroradiology, Department of Radiology Hospital Universitari Vall D'hebron Barcelona Spain; ^15^ Department of Neurology Medical University of Graz Graz Austria; ^16^ Neuroimaging Research Unit, Division of Neuroscience IRCCS San Raffaele Scientific Institute, Vita‐Salute San Raffaele University Milan Italy; ^17^ Vita‐Salute San Raffaele University Milan Italy; ^18^ Neurology Unit, Neurorehabilitation Unit, Neurophysiology Service, and Neuroimaging Research Unit, Division of Neuroscience, IRCCS San Raffaele Scientific Institute Milan Italy; ^19^ Institute of Neuroradiology, St. Josef Hospital, Ruhr‐University Bochum Bochum Germany; ^20^ Department of Neurology St. Josef Hospital, Ruhr University Bochum Bochum Germany; ^21^ Department of Neurosciences, Biomedicine and Movement Sciences University of Verona Verona Italy; ^22^ Department of Information Engineering University of Padova Padova Italy; ^23^ Department of Neurology and Center of Clinical Neuroscience First Faculty of Medicine, Charles University and General University Hospital Prague Czech Republic; ^24^ Department of Radiology First Faculty of Medicine, Charles University and General University Hospital Prague Czech Republic; ^25^ Lysholm Department of Neuroradiology UCLH National Hospital for Neurology and Neurosurgery London UK; ^26^ E‐Health Center, Universitat Oberta de Catalunya Barcelona Spain; ^27^ Dementia Research Centre, UCL Queen Square Institute of Neurology London UK

## Abstract

The brain‐predicted age difference (brain‐PAD) is associated with measures of clinical interest in people with multiple sclerosis (pwMS). Most brain age models rely on 3D T1‐weighted scans, which are not routinely acquired in MS clinical practice, limiting their potential for clinical translation. We aimed to develop a model predicting brain age using T2‐FLAIR, the core sequence for MS diagnosis and monitoring, and validate the resulting brain‐PAD values as a biomarker of MS severity and progression. We collected 3D T2‐FLAIR and 3D T1‐weighted brain MRI scans to compose (i) a multicentre cohort of healthy participants for brain age modeling, and (ii) a single‐centre cohort of pwMS and healthy controls for external validation. We trained and evaluated 3D convolutional neural network models predicting brain age from T2‐FLAIR or T1‐weighted images. Models were compared using t‐tests based on bootstrapped standard errors. Saliency maps were obtained with the SmoothGrad method to visualize regions that were most important for the predictions. Finally, using a linear model framework, we clinically validated the resulting brain‐PAD metric by assessing its relationship with diagnosis (MS versus healthy controls), clinical phenotype, disease duration, and physical disability as measured with the Expanded Disability Status Scale (EDSS), adjusting for age and sex. The Inception‐ResNet‐V2 model based on T2‐FLAIR scans yielded accurate brain age predictions (test set MAE = 3.31 years, R^2^ = 0.944, 5x ensemble MAE = 2.81, R^2^ = 0.955), which were comparable to those obtained with the T1w‐based model (test set MAE = 3.34 years, R^2^ = 0.942, 5x ensemble MAE = 2.84, R^2^ = 0.955, *p* = 0.91). Brain age predictions were mostly driven by subcortical regions, particularly the thalamus. T2‐FLAIR‐based brain‐PAD was higher in pwMS than healthy controls (7.07 vs −0.50 years, *p* < 0.0001). As with T1 brain‐PAD, FLAIR brain‐PAD correlated with MS disease duration (*R* = 0.24, *p* < 0.0001) and EDSS (*R* = 0.30, *p* < 0.0001). Brain age predictions relying on T2‐FLAIR scans are as accurate as those derived from T1‐weighted scans and could be used as an easily obtainable biomarker of MS severity and progression in clinical practice.

## Graphical abstract

While the brain‐age paradigm has emerged as a promising biomarker of neurodegeneration in multiple sclerosis (MS), most available brain‐age prediction models are based on 3D‐T1w scans, which are not acquired in routine clinical practice. Leveraging a large multicentre collection of scans from healthy controls, we developed an accurate brain‐age prediction model using 3D T2‐FLAIR scans, the core sequence for MS diagnosis and monitoring. We demonstrated that brain age predictions relying on T2‐FLAIR scans are as accurate as those derived from T1w scans and could be used as an easily obtainable biomarker of MS severity and progression in clinical practice.

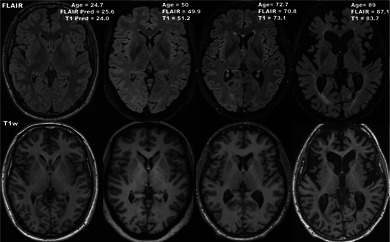

**Abbreviations**
AD: alzheimer's disease
ADNI: alzheimer's disease neuroimaging initiative
Brain‐PAD: brain predicted age difference
CNN: convolutional neural network
EDSS: expanded disability status scale
EPAD: European prevention of alzheimer's dementia
FLAIR: fluid‐attenuated inversion recovery
GM: gray matter
HC: healthy control
Inception Net: Inception‐ResNet‐V2 Network
MAE: mean absolute error
MAGNIMS: magnetic resonance imaging in MS
MNI: Montreal Neurological Institute
pwMS: people with multiple sclerosis
SFCN: simple fully convolutional network
WM: white matter

## 1 Introduction

As life expectancy increases and the burden of neurodegenerative diseases grows, understanding and quantifying age‐related brain changes and their relationship to disease become increasingly crucial (Hou et al. 2019).

**FIGURE 1:**
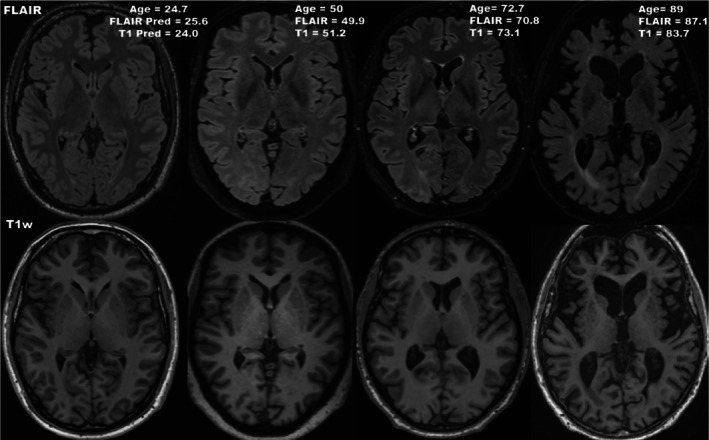
T2‐FLAIR (top) and T1w (bottom) MRIs of healthy controls of varying ages. With increasing age, the size of the ventricles can be seen to enlarge, and the sulci widen. At older ages, white matter lesions and thinning of the gray matter can be seen. Gray matter thinning can be better appreciated on T1w images, while white matter lesions are better seen on T2‐FLAIR scans. Brain‐predicted ages (Pred) are shown from our final FLAIR and T1w models combined (ensemble outputs averaged). Subjects were taken from the test set (unseen during training).

In the course of an individual's life, their brain undergoes molecular, microscopic, and macroscopic changes (Fjell and Walhovd 2010). Brain aging is associated with volume loss (approximately 0.5%–1% volume loss per year with healthy aging), enlargement of cerebrospinal fluid spaces, and white matter lesions (WML) (Fjell and Walhovd 2010). Underlying these macroscopic changes are microscopic phenomena such as axonal loss, shrinkage of neurons, and reduction of synapses, which subtend age‐related decreases in cognitive abilities (Fjell and Walhovd 2010). At the molecular level, brain aging is driven by factors such as genetic instability, DNA damage, telomere attrition, and mitochondrial dysfunction (Hou et al. 2019). Macrostructural age‐related brain changes can be visualized through structural MRI, with different contrasts/sequences potentially revealing distinct aspects of the brain's anatomy (Figure 1) (Bethlehem et al. 2022).

Although age‐related brain changes are well established, these changes affect individuals differently and at different rates, suggesting that the underlying biological age of a person's brain may be different from their chronological age (Cole and Franke 2017). Building on this concept, the brain‐age paradigm has emerged as a powerful tool to measure the brain's biological age using neuroimaging data (Cole and Franke 2017). Briefly, machine learning algorithms are used to learn a function that maps brain MRI scans onto chronological age in healthy subjects, which is then used to make individual brain age predictions on new data (Cole and Franke 2017). Among the machine‐learning techniques used for age prediction, recent years have seen growing interest in deep learning, which is potentially more powerful than shallow learning and has the advantage of being applicable to raw data (Tanveer et al. 2023). In fact, state‐of‐the‐art brain‐age prediction models are based on convolutional neural network (CNN) architectures, trained on thousands of scans from large‐scale open datasets (Bashyam et al. 2020; Peng et al. 2021). The difference between predicted and chronological age (the brain‐predicted age difference, brain‐PAD) is thought to reflect deviation from normal brain aging, indicating slower or accelerated biological aging, and has been proposed as an age‐adjusted global index of brain health (Cole and Franke 2017).

Higher brain‐PAD values are associated with other established biomarkers of aging such as weaker grip strength, slower walking speed, lower fluid intelligence, shorter leukocyte telomere length, and ultimately increased mortality risk, as well as with many different neurological, psychiatric, and systemic disorders (Cole, Ritchie, et al. 2017; Kaufmann et al. 2019). Particularly in people with multiple sclerosis (pwMS), there is robust evidence that brain‐PAD is systematically higher than in healthy controls, relates to physical disability and cognitive performance, and helps to predict disability progression (Brier et al. 2023; Cole et al. 2020; Denissen et al. 2022; Høgestøl et al. 2019; Pontillo et al. 2024; La Rosa et al. 2025). Therefore, brain‐PAD may potentially outperform other established MRI biomarkers of neurodegeneration in pwMS such as the brain parenchymal fraction (Høgestøl et al. 2022).

However, the vast majority of studies on brain age prediction and corresponding models use 3D T1‐weighted (T1w) MRI datasets, which are the most commonly available modality in research datasets but are not always acquired in routine clinical practice for pwMS, limiting the potential of the brain‐age paradigm for clinical translation. Indeed, according to current guidelines, high‐resolution T1w sequences are not mandatory for the diagnosis or monitoring of MS, unlike 3D T2‐FLAIR which is the core sequence due to its superior sensitivity in detecting lesions (Wattjes et al. 2021). Additionally, a recent audit of MRI practices in the UK showed that the majority of MS centres audited performed 3D T2‐FLAIR and only 3 performed 3D T1 for follow‐up MRIs of pwMS (Fernandes et al. 2021). In addition, T1w scans might be less sensitive than T2‐FLAIR to WML, which are an important feature of MS but do not directly influence brain age predictions with T1w‐based models (Cole et al. 2020). On the other hand, it is also possible that the higher sensitivity to WML may hinder accurate brain age predictions from T2‐FLAIR scans.

In this study, leveraging a large multicentre collection of 3D T2‐FLAIR scans from healthy controls from the MAGNIMS network, we aimed to develop an accurate brain‐age prediction model using T2‐FLAIR scans. We tested different deep learning architectures and conducted head‐to‐head comparisons with analogous T1w‐based benchmark models. Finally, we sought to determine the factors driving the model's predictions by means of explainability analyses and assessed its validity as a biomarker of disease severity in a single‐centre cohort of pwMS.

## 2 Methods

### 2.1 Participants

In this retrospective multicentre study, we collected demographics and both 3D T2‐FLAIR and 3D T1w MRI scans of healthy subjects from 14 sites, the majority of which were institutions in the MAGNetic resonance Imaging in MS (MAGNIMS) consortium (www.magnims.eu). The remaining data sources included both open datasets and other research groups upon request. For external validation in MS, we also collected clinico‐demographic and MRI data of pwMS and healthy participants from an additional MAGNIMS site (Prague).

Data from 1994 healthy participants were collected for modeling brain age, of which 1957 had both 3D T2‐FLAIR and 3D T1w MR images available and used in this study (M/F ratio: 1.36, mean age ± SD: 56.7 ± 16.2 years, age range: 18–95 years). The characteristics of the individual datasets are described in Table 1. The histogram of age distribution is shown in Figure S1. The MS validation cohort consisted of 1787 pwMS (1555 relapsing–remitting, 213 secondary progressive, 19 primary progressive) and a further 102 healthy controls (Table 2).

**TABLE 1:** Demographic and MRI characteristics of the brain age modeling cohort.

Cohorts	MR field strength (Tesla)	*N* subjects	Age range (years)	Mean age ± SD (years)	M/F Ratio	Image voxel size FLAIR (mm)	Image voxel size T1w (mm)	References
Brain age modeling cohort	1.5 & 3	1957	18–95	56.7 **±** 16.2	1.36	Resampled to 1 × 1 × 1	Resampled to 1 × 1 × 1	~
ADNI	3	100	58–91	76.5 **±** 8.0	1.04	1.2 × 1 × 1	1 × 1 × 1	1
Barcelona	3	53	23–61	45.2 **±** 9.5	2.31	1 × 1 × 1	1 × 1 × 1	2
Bochum	3	49	19–65	39.2 **±** 13.5	3.1	1 × 1 × 1	1 × 1 × 1	2
EPAD	1.5 & 3	886	50–88	64.3 **±** 6.9	1.39	1 × 1 × 1	1.2 × 1.06 × 1.06	3
Graz	3	34	21–52	30.3 **±** 8.9	1.43	1 × 1 × 1	0.8 × 0.8 × 0.8	2
Hanover	3	14	41–71	52.1 **±** 8.6	0.4	0.9 × 0.51 × 0.51	0.9 × 1.02 × 1.02	2
Mainz	3	57	20–57	30.7 **±** 9.9	1.19	1 × 0.5 × 0.5	1 × 1 × 1	2
Milan	3	189	18–77	33.9 **±** 11.8	0.89	0.89 × 0.89 × 1	1 × 1 × 1	2
Naples	3	61	24–64	43.0 **±** 11.2	0.45	1 × 1 × 1	1 × 1 × 1	4
NIFD	3	129	36–81	63.0 **±** 7.6	1.3	1 × 0.98 × 0.98	1 × 1 × 1	5
NORMENT	3	315	21–95	59.2 **±** 14.8	1.69	1.2 × 1 × 1	1 × 1 × 1	6
Oslo	3	24	20–51	34.7 **±** 8.6	1.4	1.2 × 1 × 1	1 × 1 × 1	2
Oxford	3	11	24–58	35.9 **±** 13.6	0.83	1 × 1 × 1.05	1 × 1 × 1	2
Siena	3	27	25–56	36.0 **±** 8.6	0.8	1 × 0.98 × 0.98	1 × 1 × 1	2

*Note:* References 1 = (ADNI 2017), 2 = (Group 2021), 3 = (Solomon et al. 2018), 4 = (Tranfa et al. 2025), 5 = (Boeve et al. 2021), 6 = (Nerland et al. 2022).

Abbreviations: ADNI, alzheimer's disease neuroimaging initiative; EPAD, european prevention of alzheimer's dementia.

**TABLE 2:** Demographic and clinical characteristics of the MS validation cohort.

	pwMS	HC
*N*	1787	102
Mean age (years)	45.2 ± 8.97	40.7 ± 10.66
M/F (N)	493/1294	36/66
Mean disease duration (years)	13.8 ± 8.98	—
Mean EDSS	2.62 ± 1.36	—
RR/PP/SP (N)	1555/19/213	—
Disease‐modifying therapy, first line/second line/none (%)	50.3/34.6/15.1	—
T1w acquisition details	3T Siemens Skyra: TR = 2300ms, TE = 3ms, TI = 900ms, FA = 9°, 176 sagittal slices	
FLAIR acquisition details	3T Siemens Skyra: TR = 5000ms, TE = 397ms, TI = 1800ms, FA = 120°, 176 sagittal slices	

Abbreviations: EDSS, expanded disability status scale; HC, healthy controls; PP, primary progressive; pwMS, people with multiple sclerosis; RR, relapsing‐remitting; SP, secondary progressive.

The project was carried out in accordance with the Declaration of Helsinki. Written informed consent was obtained from each participant independently at each centre. The final protocol for this study was reviewed and approved by the local ethics committees (project ID number 19143/001) and the MAGNIMS Study Group Steering Committee.

### 2.2 Brain Age Modeling

Three 3D CNN architectures recently used to obtain state‐of‐the‐art performance in brain age prediction were trained and evaluated on the healthy control data: a simple fully convolutional network (SFCN) (Peng et al. 2021), a DenseNet‐169 (Wood et al. 2022), and a 3D Inception‐ResNet‐V2, referred to as InceptionNet in this work (Bashyam et al. 2020). These architectures have been previously trained from scratch (without pre‐training) for brain age prediction tasks on training datasets of a comparable size to ours, showing good performances (Cole, Poudel, et al. 2017; Montella et al. 2024). Additionally, when available (i.e., for the SFCN), we used pre‐trained weights to initialise the model, ensuring smooth adaptation to relatively smaller training data. Details on the models' architecture and the training/validation/testing procedures are provided in the Data S1. Briefly, MR images underwent minimal preprocessing including intensity inhomogeneity correction using the N4 method and affine registration to the MNI152 template. Alternative preprocessing pipelines were also evaluated, including rigid (rather than affine) registration to the MNI152 template or using skull‐stripped (rather than whole‐head) volumes. Before being presented to the models, images were resampled/cropped to reduce array size and computational burden while retaining anatomical details, normalised by subtracting the mean and dividing by the standard deviation, and scaled to make voxel values vary between 0 and 1. Online data augmentation was also performed, including random spatial and intensity transformations, with the goal of making the network invariant to image quality variations and site effects. After randomly splitting the brain age modeling cohort, both T2‐FLAIR and T1w‐based models were trained on 80% of the subjects, with 10% left to evaluate the training progress and select the epoch with the highest accuracy (validation set). The best models were then evaluated on the remaining 10% unseen subjects (test set), with performance quantified in terms of mean absolute error (MAE), correlation coefficient (R), and coefficient of determination (R^2^). The best‐performing CNN architecture was selected for further analyses. To reduce the variance in the predictions, we trained the same architecture ten times, combined the five models that performed best on the validation data set, and used the mean output as the final prediction (model averaging ensemble).

### 2.3 Age Bias

The final model was used to assess age bias (i.e., the overestimation of younger subjects' brain age and underestimation of older subjects' brain age), quantified as the correlation coefficient between brain‐PAD and chronological age in the validation set. Age predictions on the validation set were also used to estimate a linear fit between predicted and chronological age, which was then used to correct external predictions for age bias (de Lange and Cole 2020).

### 2.4 Model Interpretability

To identify the main drivers behind the models' predictions, we obtained subject‐level saliency maps on the test set using the SmoothGrad method (Smilkov et al. 2017), which has previously been used in the brain age prediction context^19^. Maps were aggregated by nonlinearly registering individual maps to the MNI152 template, smoothing with a 6 mm FWHM Gaussian kernel, and averaging values across subjects (Levakov et al. 2020). To anatomically contextualize the maps, the mean saliency and number of highly salient voxels (i.e., top 1% saliency) were computed for GM and WM regions of the DKT and ICBM DTI‐81 atlases, respectively (Levakov et al. 2020).

Also, to assess the possible impact of WML on age predictions, we automatically segmented WML on the scans of healthy subjects of the test set (Valverde et al. 2017) and artificially removed them using an inpainting algorithm (Prados et al. 2016) to obtain lesion‐filled scans. Then, we compared age predictions obtained from raw and lesion‐filled scans.

### 2.5 Statistical Analysis

Statistical analyses were performed, and plots produced, using R (Version 4.1.1). To compare the Mean Absolute Error (MAE) of different brain age models, the Standard Error (SE) was calculated using bootstrapping with 1000 samples, using the R package ‘boot’ (Davison and Hinkley 1997). The standard deviation (SD) was computed by multiplying the SE by $\sqrt{N}$ (where N is the number of testing subjects, in this case 174) and used to perform two‐tailed T‐tests. In the MS validation cohort, we assessed possible between‐group differences (pwMS vs. healthy controls, RR vs. SP vs. PP) in terms of brain‐PAD with ANCOVA analyses, adjusting for age and sex. The associations between brain‐PAD values and MS severity (as measured with the Expanded Disability Status Scale (EDSS)) and duration, as well as with other established measures of disease severity (namely, total lesion volume—TLV—and brain parenchymal fraction—BPF), were assessed with partial correlation analyses correcting for age and sex.

The Shapiro–Wilk Test for normality was performed to check the distributions of variables before all parametric tests. Bonferroni correction for family‐wise error was performed on all *p*‐value thresholds when testing for significance to keep the false positive rate at 0.05 for each set of tests.

## 3 Results

### 3.1 Brain Age Prediction

The three 3D CNN models were trained, validated, and tested on 1394/174/174 participants respectively, as 215 subjects with multiple time points were excluded from model training. Prediction accuracies on the test set are shown in Table 3. When using T2‐FLAIR scans, the Inception‐ResNet‐V2 model yielded the highest accuracy (MAE = 3.31 years, R^2^ = 0.944), outperforming the SFCN model (MAE = 4.12, R^2^ = 0.916, *p* = 0.0097) although not a statistically significant better accuracy compared to the DenseNet (MAE = 3.74, R^2^ = 0.921, *p* = 0.15) model. For T1w‐based models, the highest accuracy was also achieved with the Inception‐ResNet‐V2 architecture (MAE = 3.34 years, R^2^ = 0.942), although with no significant difference between architectures (*p* = 0.76 and 0.73 for the comparisons with the DenseNet and the SFCN models, respectively). There was no significant difference between FLAIR and T1w‐based models for the best‐performing architecture (*p* = 0.91), with a high correlation between the corresponding age predictions (R^2^ = 0.937).

**TABLE 3:** Performance of the 3 CNN models in the test set, evaluated on FLAIR and T1w scans.

Model	FLAIR				T1w			
	MAE	R	R^2^	*p*	MAE	R	R^2^	*p*
Inception‐ResNet‐V2	3.31 **±** 2.44	0.972	0.944	~	3.34 **±** 2.47	0.971	0.942	0.91
DenseNet169	3.74 **±** 3.14	0.960	0.921	0.15	3.43 **±** 2.98	0.965	0.931	0.76
SFCN (pretrained)	4.12 **±** 3.31	0.957	0.916	**0.0097**	3.45 **±** 3.31	0.965	0.931	0.73

*Note:* The *p* value for T1w Inception‐ResNet‐V2 model based on the comparison with the FLAIR Inception‐ResNet‐V2 model. All remaining *p*‐values for DenseNet169 and SFCN models are based on the comparison with the Inception‐ResNet‐V2 model using the same modality. Bootstrapping with 1000 samples was used to derive standard errors to perform t‐tests. For statistically significant comparisons, *p*‐values are reported in bold.

Abbreviation: MAE, mean absolute error.

When evaluating the impact of different preprocessing strategies on the model performance, there were no significant effects of the preprocessing pipeline, except for a decrease in accuracy when using skull‐stripped T1w images (MAE = 4.44 years, R^2^ = 0.896, *p* = 0.00051) (Table S1).

Ensemble averaging of Inception‐ResNet‐V2 models (i.e., trained 5 times with random initialization of the weights) yielded an improvement in accuracy when relying on the FLAIR, T1, or FLAIR+T1 predictions (Table S2). There was no significant performance difference across modalities (MAEs of 2.81 and 2.84 years for the FLAIR x5 and T1w x5 ensembles, respectively). A combination of FLAIR and T1w, by averaging the predictions, yielded an improvement in performance, although this was not significant (MAE = 2.57, *p* = 0.30) (Figure S4). Based on these results, the FLAIR x5 and T1w x5 ensembles, as well as their combination, were selected as the final models for brain age prediction.

#### 3.1.1 Age Bias

**FIGURE 2:**
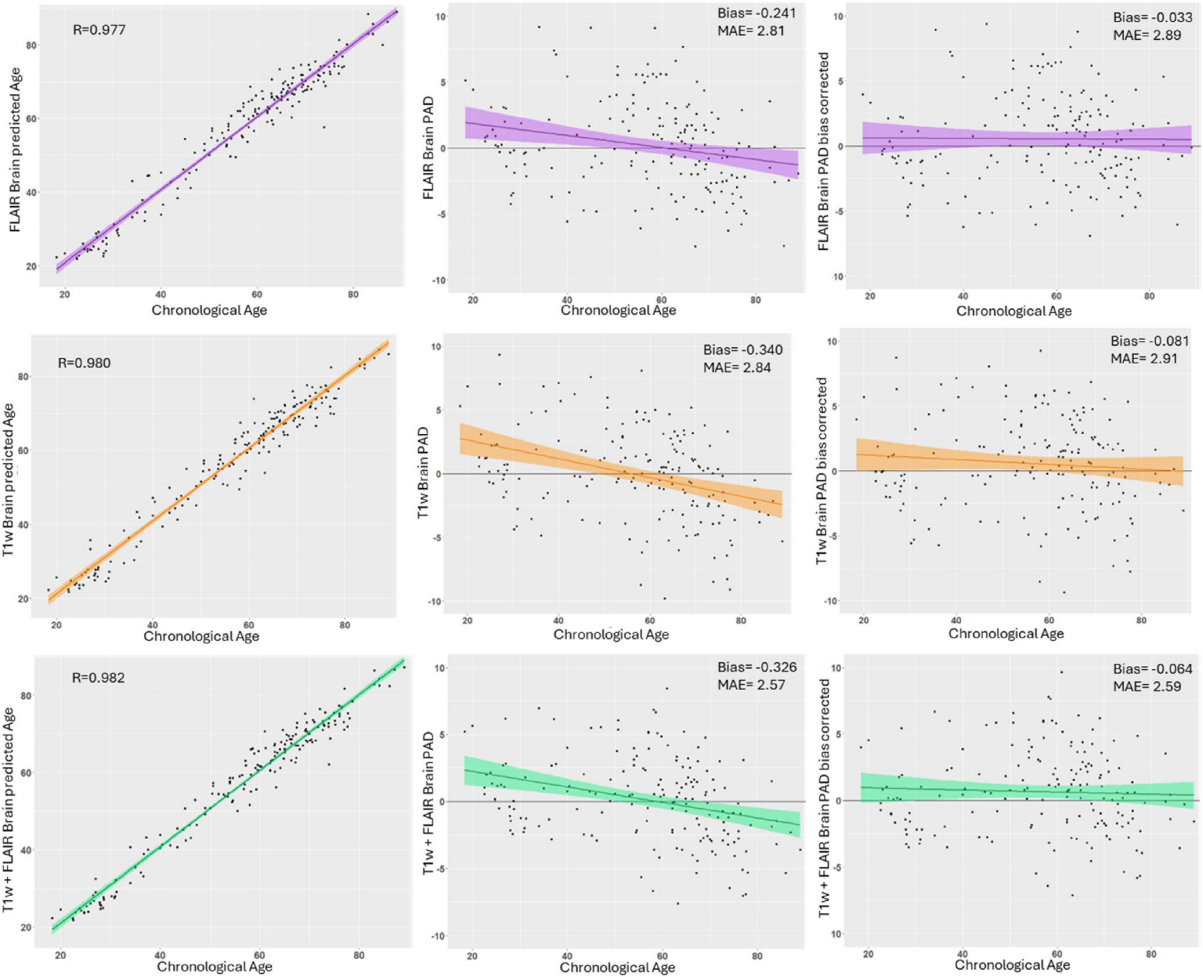
Scatter plots showing the predictive performance and the age bias of the models. Correlations of brain‐predicted age with chronological age are shown in the left column. The other columns display the correlations between brain‐PAD and chronological age, before (middle column) and after (right column) bias correction. The FLAIR‐based model is shown in the top row, the T1w‐based model in the middle row, and the combined T1w + FLAIR model in the bottom row. MAE = mean absolute error. Bias is defined as the Pearson correlation coefficient between chronological age and brain‐PAD.

Both the FLAIR and T1w models tended to overestimate brain age in younger subjects and vice versa, with biases (i.e., the correlation between chronological age and brain‐PAD) of −0.241 and −0.340, respectively (Figure 2). The bias correction procedure greatly reduced the bias of the FLAIR and T1w models to −0.033 and −0.081 for the FLAIR and T1w models, respectively, while only marginally reducing the models' accuracies (MAE increasing from 2.81 to 2.89 for the FLAIR model and from 2.84 to 2.91 for the T1w model) (Figure 2).

#### 3.1.2 Model Interpretability

**FIGURE 3:**
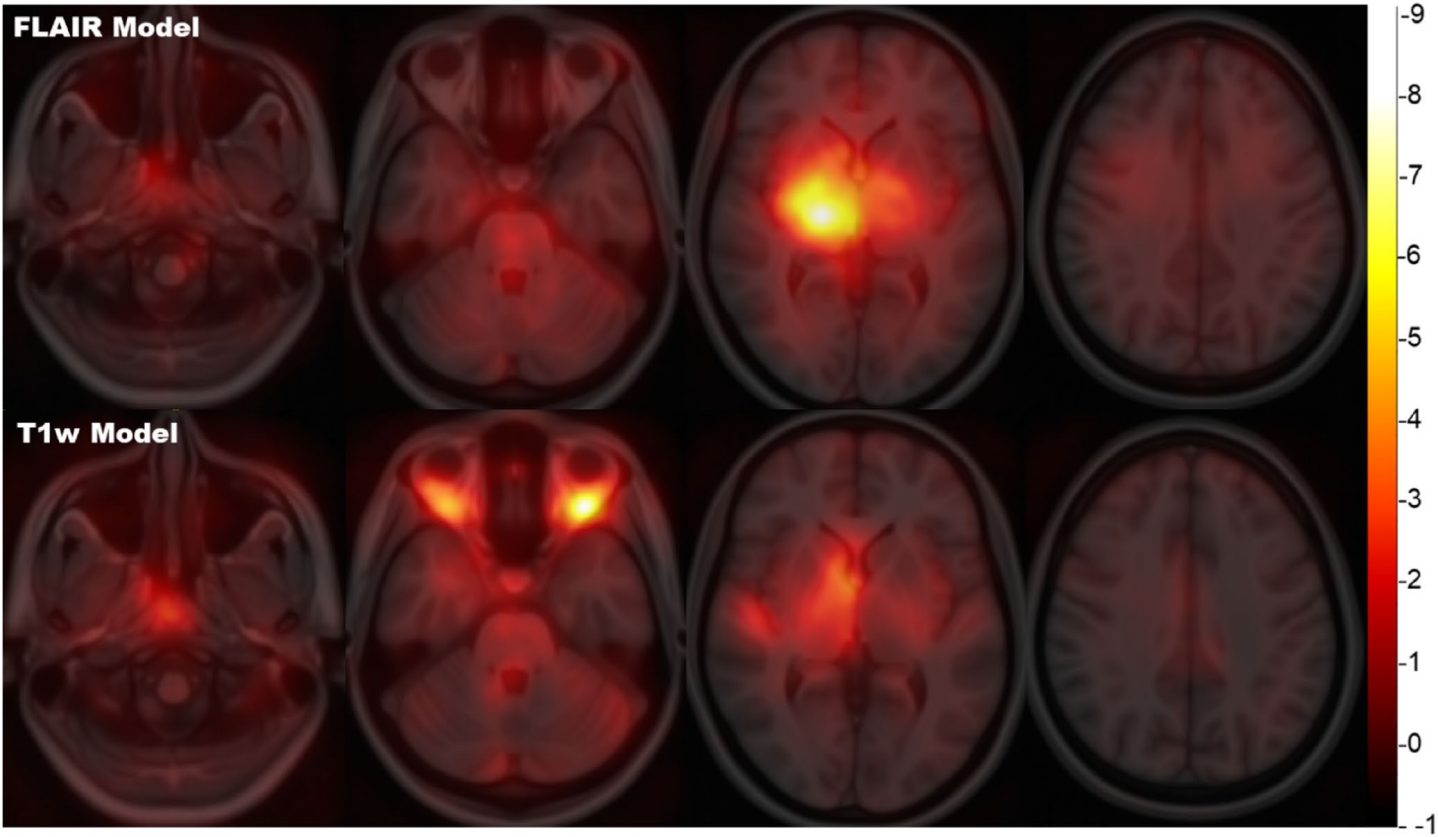
Group average saliency maps. Saliency maps for FLAIR‐based (top) and T1w‐based (bottom) InceptionNet models, normalized to the MNI space, smoothed, and averaged across subjects. Unthresholded maps are superimposed on the MNI brain template (scale displayed on the right).

Group‐averaged saliency maps from the test set for the FLAIR and T1w models are shown in Figure 3. The mean saliency and number/proportion of highly salient voxels for the most influential regions are presented in Tables S3 and S4. For both modalities, the model predictions were mostly driven by regions within the brain, with a particular focus on areas such as the ventricles, thalamic/diencephalic and nucleo‐capsular structures, and the brainstem. However, high saliency was also observable in cervicofacial regions, with a prominent role for retroorbital fat in the T1w model, suggesting that areas outside the brain may also contain age‐related information, especially from a T1 contrast. Similar brain regions were highlighted in saliency maps obtained for models trained on skull‐stripped images (Figure S6).

As for the impact of WML, there were 2.6 ± 19.4 lesions on average per subject, with a mean lesion volume of 10,550 ± 16,147 mm^3^ in the 174 subjects of the test set. An example of a lesion‐filled FLAIR scan is shown in Figure S5. Age predictions obtained with the Inception‐ResNet‐V2 FLAIR model on lesion‐filled scans did not significantly differ from those based on raw images (MAE lesion filled: 3.35 vs. raw: 3.31, *p* = 0.88), suggesting that WML did not contribute to brain age estimation (Table S5).

### 3.2 Validation in Multiple Sclerosis

**FIGURE 4:**
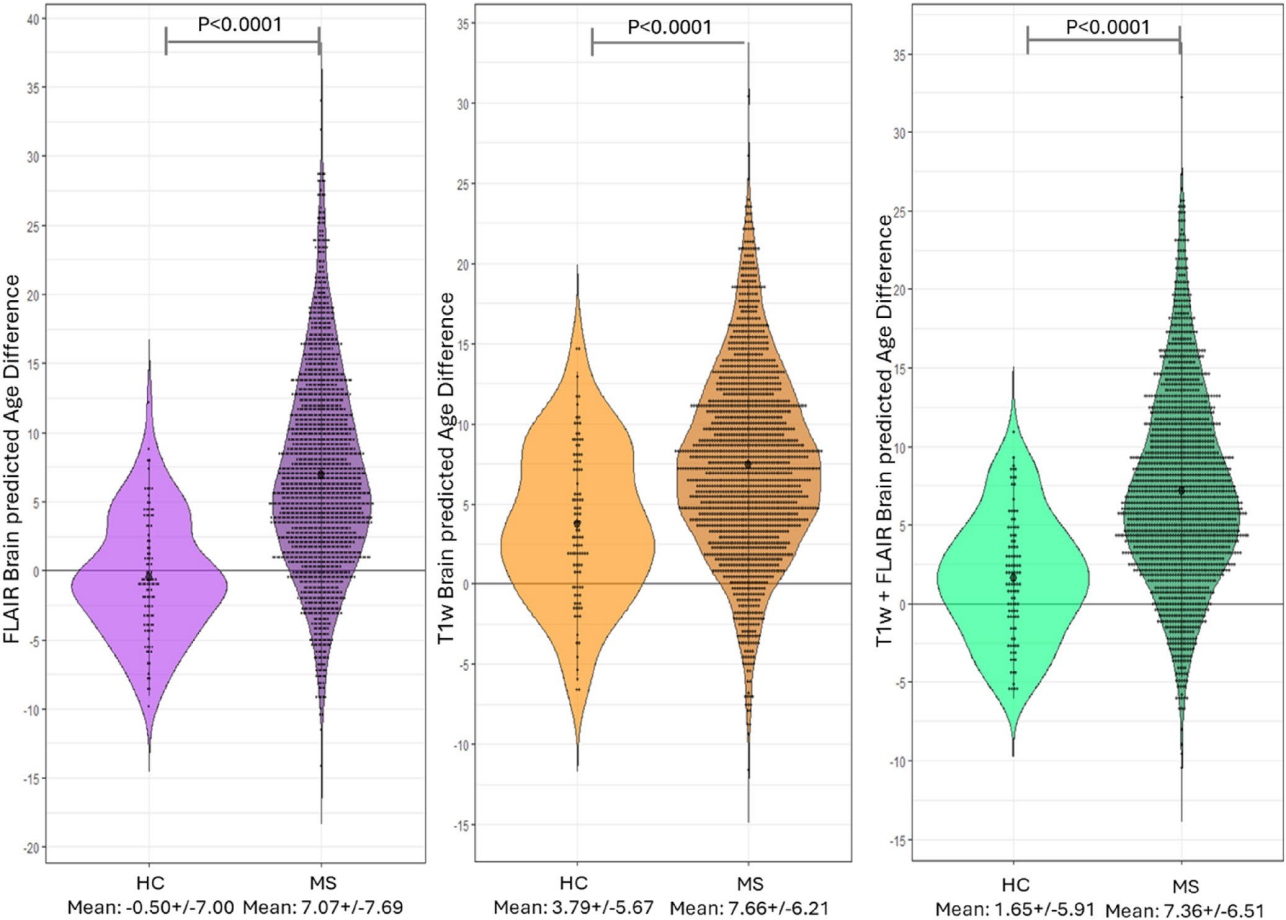
Between‐group comparison in terms of Brain‐PAD. Box plots of Brain‐PAD for pwMS (MS) and healthy controls (HC) of the validation cohort, for the FLAIR, T1w, and FLAIR + T1w InceptionNet models (from left to right).

**FIGURE 5:**
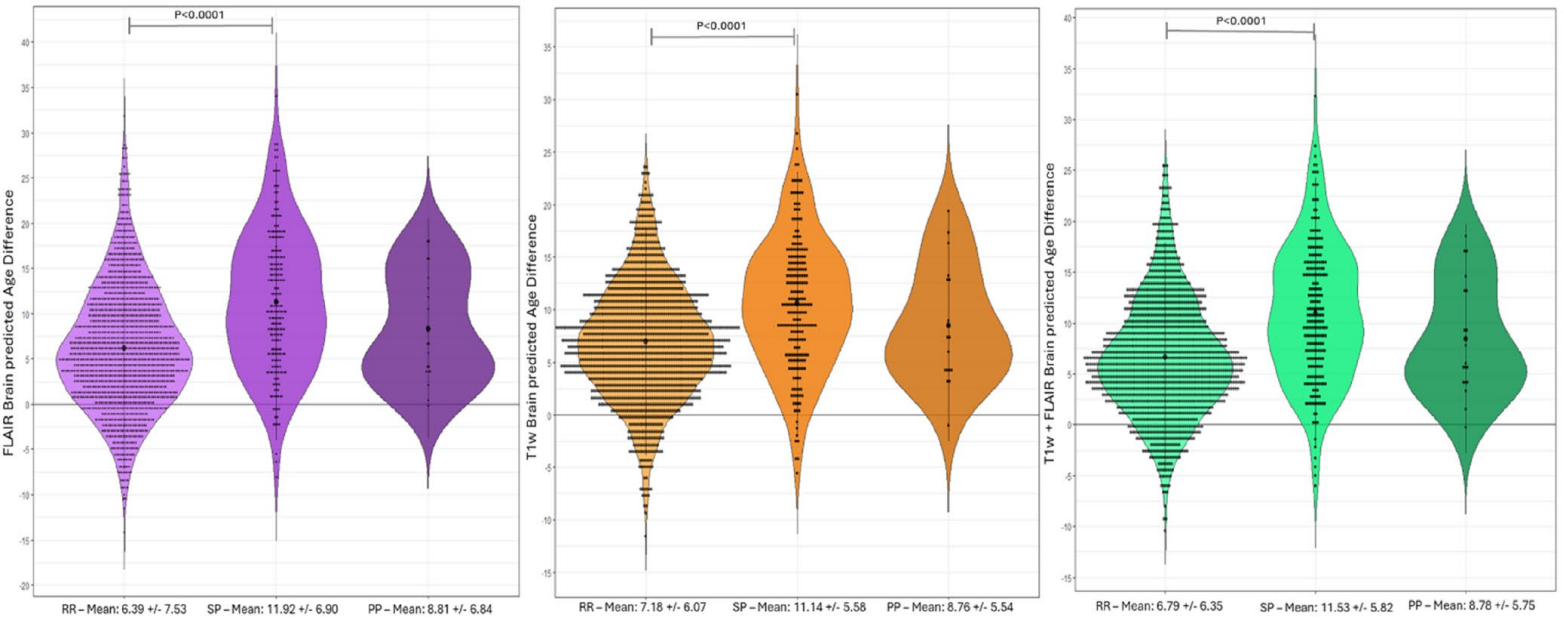
Box plots of Brain‐PAD for MS subtypes (RR‐Relapsing–remitting, SP‐Secondary progressive, PP‐Primary progressive) for FLAIR, T1w, and FLAIR + T1w InceptionNet models (from left to right).

The 3D Inception‐ResNet‐V2 model remained accurate with healthy controls from the independent validation cohort, which was not included in the training and evaluation procedure, with MAEs of 3.47 and 4.86 for the FLAIR and T1w models respectively. When analyzing the brain‐PAD values of pwMS, their brains looked significantly older on average than those of healthy controls (age‐ and sex‐adjusted mean brain‐PAD = FLAIR: 7.07 vs −0.50 years, *p* < 0.0001; T1w: 7.66 vs 3.79 years, *p* < 0.0001) (Figure 4). The brain‐PAD metric was also sensitive to clinical phenotype (*p* < 0.0001), with patients with secondary progressive forms showing higher values compared to patients with a relapsing remitting disease course (adjusted mean brain‐PAD = FLAIR: 11.92 vs. 6.39 years, *p* < 0.0001; T1w: 11.14 vs. 7.18 years, *p* < 0.0001). No significant differences emerged when comparing primary progressive patients with either secondary progressive or relapsing remitting forms (Figure 5), however there was a significant difference between brain‐PAD of the relapsing remitting subtype and progressive forms (adjusted mean brain‐PAD = FLAIR: 6.39 vs. 11.67, *p* < 0.0001; T1w: 7.18 vs. 10.95, *p* < 0.0001).

**FIGURE 6:**
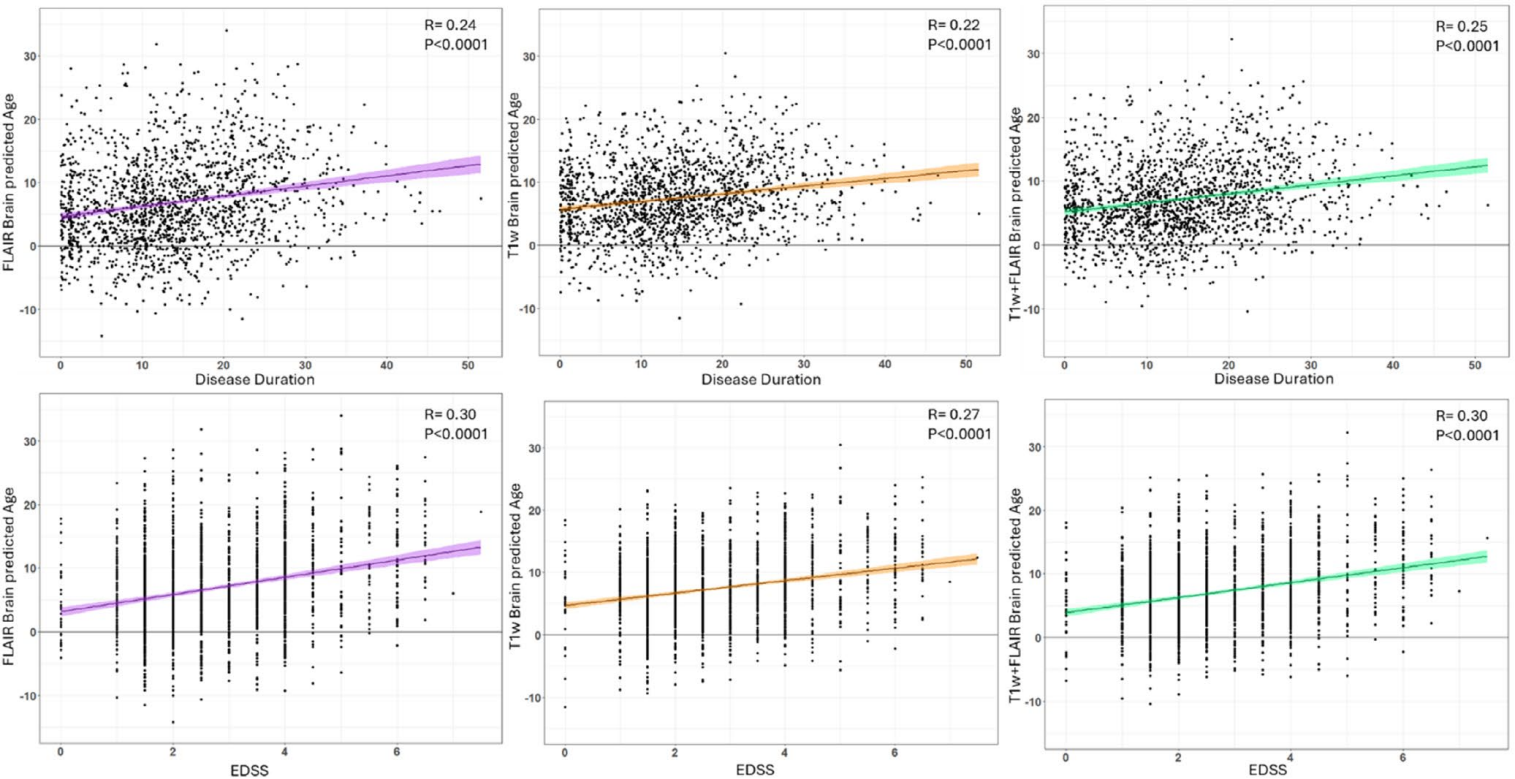
Validation of the brain‐PAD metric in the MS cohort. Scatter plots of Brain‐PAD and disease duration (years) in the top row and Brain‐PAD and EDSS in the bottom row for FLAIR, T1w, and FLAIR + T1w models (from left to right). Correlation analysis adjusted for age and gender as covariates.

Brain‐PAD was also significantly associated with disease duration and severity (using linear models with age and gender as covariates), with older appearing brains corresponding to longer disease lengths (FLAIR: *R* = 0.24, *p* < 0.0001; T1w: *R* = 0.22, *p* < 0.0001) and higher EDSS scores (FLAIR: *R* = 0.30, *p* < 0.0001; T1w: *R* = 0.27, *p* < 0.0001) (Figure 6). Both FLAIR‐ and T1w‐based brain‐PAD values were found to correlate with TLV (0.610 and 0.475, respectively, *p* < 0.0001) and BPF (−0.600 and −0.493, respectively, *p* < 0.0001).

## 4 Discussion

Leveraging a large multicentre MRI dataset, we developed an accurate brain‐age model relying on 3D T2‐FLAIR images that have potential for clinical applicability in MS.

The model was based on a 3D version of the Inception‐ResNet‐v2, which slightly outperformed the state‐of‐the‐art DenseNet169 and SFCN architectures. We showed that the 3D T2‐FLAIR and T1w‐based models achieved comparable accuracies, suggesting a similar amount of age‐related information is contained in the two sequences. Interestingly, averaging the T2‐FLAIR and T1w predictions slightly improved the model's accuracy further. This shows that the two sequences also yield complementary, contrast‐specific, information, and highlight the potential of multimodal imaging for brain age prediction (Rokicki et al. 2021). The choice of the preprocessing strategy did not appear to influence the models' accuracies, except for a small detrimental effect of skull‐stripping on T1w‐based brain age predictions, potentially due to removal of CSF spaces or extracranial tissues.

As for all brain age prediction models, our model was also prone to age bias, that is, the overestimation of age in younger subjects and vice versa, which was effectively reduced with minimal accuracy loss using a linear correction model. This highlights once again the importance of applying an adequate statistical bias correction before or during downstream analyses (de Lange and Cole 2020). In line with previous evidence on state‐of‐the‐art T1w‐based brain age models (Dörfel et al. 2023), our model showed excellent test–retest reliability in healthy controls, demonstrating its robustness to acquisition‐related, non‐biologically relevant factors.

When looking at what was driving brain age predictions, the 3D T2‐FLAIR model was shown to use plausible imaging features. Saliency maps highlighted structures encompassing the deep grey matter nuclei and the ventricles, possibly pointing at age‐related subcortical volume loss. The thalamus was the most salient structure, which is potentially explained by previous evidence showing a consistently linear volume loss of the thalamus with age, as opposed to age‐related cortical thinning which can be more heterogenous (Bethlehem et al. 2022). Interestingly, the comparison between model predictions obtained on raw versus lesion‐filled brain scans showed that the presence of WML did not significantly influence age estimation. Indeed, the incidence and load of WML associated with healthy ageing are relatively low (Kim et al. 2008), which substantiates the marginal weight attributed to this feature by brain age prediction models. This observation also suggests that MS‐related demyelinating lesions are unlikely to skew the model's predictions too heavily, as shown in previous studies (Cole et al. 2020; Pontillo et al. 2024). Of note, the T1w model additionally made use of the signal from areas outside the brain such as the orbits, which may be explained by age‐related changes in body composition and adipose tissue characteristics (Beck et al. 2024; Darcy et al. 2008; Ou et al. 2022; Ponti et al. 2019), which are best captured on the T1 contrast.

We externally validated the model on a large MS dataset, corroborating an expanding body of evidence that demonstrates the association between the brain‐PAD metric and measures of clinical interest in MS (Brier et al. 2023; Cole et al. 2020; Høgestøl et al. 2019; Pontillo et al. 2024). Importantly, we showed higher brain‐PAD values in MS patients compared to healthy controls for both the 3D T2‐FLAIR and the 3D T1w‐based models. The brain‐PAD metric was also sensitive to clinical phenotype, with the brains of patients with secondary progressive MS appearing significantly older than those of patients with a relapsing–remitting form on both sequences. Brain‐PAD was additionally shown to correlate with disease severity and duration, with higher values associated with longer disease length and higher EDSS scores. Taken together, these results show that T2‐FLAIR‐based brain age predictions are at least non‐inferior to more established T1w‐based models as a biomarker of MS severity and progression, with the advantage of being more readily available in clinical practice. Additionally, due to the complementary information provided by the two contrasts, both models could be used in conjunction where possible. Practical clinical utility is further enhanced by the reliance on minimally pre‐processed images, which makes the prediction models faster and less susceptible to bias from pre‐processing decisions (Baecker et al. 2021). Also, having trained the model on a large and diverse dataset including data from many different centres enhances its potential generalisability, as evidenced by its high accuracy on the healthy control data from the external validation cohort.

Some limitations of this work should be acknowledged. First, most of the training data was acquired on 3 T MRI scanners, and it is unclear whether the models would be less accurate on the more common 1.5 T scanner images, potentially limiting their generalizability to real‐world scenarios. Additionally, while our brain age model was accurate and captured biologically plausible information in the MRI data, additional steps will be needed to drive the proposed approach toward clinical applicability in a real‐world setting, including the estimation of the uncertainty associated with the predictions (Faghani et al. 2023; Wei et al. 2023), and the demonstration that the proposed metric adds information above and beyond other established MRI‐derived biomarkers of neurodegeneration (e.g., global and regional brain volumes), ultimately resulting in a positive impact on the management of patients. Indeed, it is likely that brain age estimation is strongly influenced by diffuse brain atrophy, as shown in previous work (Cole et al. 2020; Pontillo et al. 2024) and hinted at by the strong correlation of both FLAIR‐ and T1w‐derived brain‐PAD values with BPF. However, while systematically investigating the additional information conveyed by brain age estimation over measures of global brain atrophy was beyond the scope of our work and would require dedicated study designs, the observation that FLAIR‐ and T1w‐derived brain‐PAD values correlated more strongly with each other than with BPF, as well as the saliency maps highlighting specific regions that are known to be prominently involved in MS, suggest that they are capturing additional information that is not already accounted for by global brain atrophy. Our model utilized minimally pre‐processed images which were not skull stripped in order to retain all potentially useful information (e.g., extra‐axial CSF spaces) and use scans in the rawest possible form. However, a limitation linked to this approach is that the models could potentially be using information from outside the brain for age prediction. An additional limitation is the relatively small size of our dataset and testing subset. Also, while the MS validation cohort was relatively large and clinically diverse, it was drawn from a single centre, which could limit the generalizability of our findings. Future work with larger (independent) test sets and/or cross‐validation (Bradshaw et al. 2023), as well as with multi‐centre clinical validation, could provide more robust estimates of the models' performance and generalizability. Also, as recent preliminary research suggests that the brain's biological age is influenced by MS‐related treatment (McMurran et al. 2025), future studies applying the brain age paradigm in a longitudinal setting will be important to clarify the effects of disease modifying therapies on the brain‐PAD metric.

In conclusion, we developed and validated an accurate 3D T2‐FLAIR brain age prediction model that is comparable to a T1w model, captures biologically plausible features from the images, and is related to disease duration and severity in pwMS, representing a viable solution for biomarker quantification in clinical practice.

## Conflicts of Interest

Massimo Filippi is a handling editor of Human Brain Mapping and a co‐author of this article. To minimize bias, they were excluded from all editorial decision‐making related to the acceptance of this article for publication.

G. Pontillo received research grants from ECTRIMS‐MAGNIMS and ESNR.

O. Goodkin is an employee of Bayer Plc.

M.A. Foster has received speaker honoraria from Merck.

M. Calabrese received speaker honoraria, consulting fees, and travel support from Biogen, Bristol Myers Squibb, Celgene, Sanofi, Merck Serono, Novartis, and Roche and received research support from the Progressive MS Alliance, the Italian Minister of Health, Roche, Novartis and Bristol Myers Squibb.

J. Palace has received support for scientific meetings and honoraria for advisory work from Merck Serono, Novartis, Chugai, Alexion, Roche, Medimmune, Argenx, Vitaccess, UCB, Mitsubishi, Amplo, Janssen. Grants from Alexion, Argenx, Roche, Medimmune, Amplo biotechnology. Patent ref P37347WO and licence agreement Numares multimarker MS diagnostics shares in AstraZeneca. Her group has been awarded an ECTRIMS fellowship and a Sumaira Foundation grant to start later this year. A Charcot fellow worked in Oxford from 2019 to 2021. She acknowledges partial funding to the trust by Highly Specialised Services NHS England. She is on the medical advisory boards of the Sumaira Foundation and MOG project charities, is a member of the Guthy Jackson Foundation Charity and is on the Board of the European Charcot Foundation and the steering committee of MAGNIMS and the UK NHSE IVIG Committee and chairman of the NHSE neuroimmunology patient pathway and ECTRIMS Council member on the educational committee since June 2023. On the ABN advisory groups for MS and neuroinflammation.

S. Messina received honoraria for lecturing and advisory board activity from UCB and Biogen, and a travel grant from Roche and Merck.

R. Cortese received speaker honoraria and travel support from Roche, Merck, Sanofi‐Genzyme, Novartis and Janssen. She was awarded a MAGNIMS‐ECTRIMS fellowship in 2019.

M. Filippi is Editor‐in‐Chief of the Journal of Neurology, Associate Editor of Human Brain Mapping, Neurological Sciences, and Radiology; received compensation for consulting services from Alexion, Almirall, Biogen, Merck, Novartis, Roche, Sanofi; speaking activities from Bayer, Biogen, Celgene, Chiesi Italia SpA, Eli Lilly, Genzyme, Janssen, Merck‐Serono, Neopharmed Gentili, Novartis, Novo Nordisk, Roche, Sanofi, Takeda, and TEVA; participation in Advisory Boards for Alexion, Biogen, Bristol‐Myers Squibb, Merck, Novartis, Roche, Sanofi, Sanofi‐Aventis, Sanofi‐Genzyme, Takeda; scientific direction of educational events for Biogen, Merck, Roche, Celgene, Bristol‐Myers Squibb, Lilly, Novartis, Sanofi‐Genzyme; he receives research support from Biogen Idec, Merck‐Serono, Novartis, Roche, the Italian Ministry of Health, the Italian Ministry of University and Research, and Fondazione Italiana Sclerosi Multipla.

E.A. Høgestøl received honoraria for lecturing and advisory board activity from Biogen, Merck and Sanofi‐Genzyme and an unrestricted research grant from Merck.

C. Lukas received a research grant from the German Federal Ministry for Education and Research, BMBF, German Competence Network Multiple Sclerosis (KKNMS, grant no. 01GI1601I) and has received consulting and speaker's honoraria from Biogen Idec, Bayer Schering, Daiichi Sanykyo, Merck Serono, Novartis, Sanofi, Genzyme and TEVA.

M.A. Rocca received consulting fees from Biogen, Bristol Myers Squibb, Eli Lilly, Janssen, Roche; and speaker honoraria from AstraZeneca, Biogen, Bristol Myers Squibb, Bromatech, Celgene, Genzyme, Horizon Therapeutics Italy, Merck Serono SpA, Novartis, Roche, Sanofi and Teva. She receives research support from the MS Society of Canada, the Italian Ministry of Health, the Italian Ministry of University and Research, and Fondazione Italiana Sclerosi Multipla. She is Associate Editor for Multiple Sclerosis and Related Disorders.

M.V. received speaker honoraria, consultant fees and travel expenses from Biogen Idec, Novartis, Roche, Merck and Teva and has been supported by the Czech Ministry of Education—project Cooperatio LF1, research area Neuroscience, and the project National Institute for Neurological Research (Programme EXCELES, ID project No LX22NPO5107)–funded by the European Union – Next Generation EU and Czech Ministry of Health– the institutional support of the research RVO VFN 64165.

A. Rovira serves/ed on scientific advisory boards for BMS, Novartis, Sanofi‐Genzyme, Synthetic MR, Tensor Medical, Roche, Biogen, and OLEA Medical, and has received speaker honoraria from Bayer, Sanofi‐Genzyme, Merck‐Serono, Teva Pharmaceutical Industries Ltd, Novartis, Roche, BMS and Biogen.

A. Toosy has received speaker honoraria from Merck, Biomedia, Sereno Symposia International Foundation, Bayer and At the Limits and meeting expenses from Merck, Biogen Idec and Novartis He was the UK PI for two clinical trials sponsored by MEDDAY pharmaceutical company (MD1003 in optic neuropathy (MS‐ON‐NCT02220244) and progressive MS (MS‐SPI2‐NCT02936037)). He has been supported by recent grants from the MRC (MR/S026088/1), NIHR BRC (541/CAP/OC/818837) and RoseTrees Trust (A1332 and PGL21/10079). He is an associate editor for Frontiers in Neurology—Neuro‐ophthalmology section and on the editorial board for Neurology and Multiple Sclerosis Journal.

O. Ciccarelli is an NIHR Research Professor (RP‐2017‐08‐ST2‐004); acts as a consultant for Biogen, Merck, Novartis, Roche, and Teva; and has received research grant support from the MS Society of Great Britain and Northern Ireland, the NIHR UCLH Biomedical Research Centre, the Rosetree Trust, the National MS Society, and the NIHR‐HTA.

F. Barkhof: Steering committee and iDMC member for Biogen, Merck, Roche, EISAI. Consultant for Roche, Biogen, Merck, IXICO, Jansen, Combinostics. Research agreements with Novartis, Merck, Biogen, GE, Roche. Co‐founder and shareholder of Queen Square Analytics LTD. The remaining authors report no competing interests.

J.H. Cole is a scientific advisor to and shareholder in BrainKey and Claritas HealthTech PTE.

## Coinvestigators

Authors are members of the MAGNIMS network (Magnetic Resonance Imaging in multiple sclerosis; https://www.magnims.eu/), which is a group of European clinicians and scientists with an interest in undertaking collaborative studies using MRI methods in multiple sclerosis, independent of any other organization. The group is run by a steering committee whose members are:

Frederik Barkhof, MD, PhD (Amsterdam), Nicola de Stefano, MD, PhD (Siena), Jaume Sastre‐Garriga, MD, PhD Co‐Chair (Barcelona), Olga Ciccarelli, MD, PhD (London), Christian Enzinger, MD, PhD (Graz), Massimo Filippi, MD, PhD (Milan), Claudio Gasperini, MD, PhD (Rome), Cristina Granziera, MD, PhD (Basel), Gabriele Deluca, MD, PhD (Oxford), Menno M. Schoonheim, PhD (Amsterdam), Alex Rovira, MD, PhD (Barcelona), Maria A. Rocca, MD, PhD Co‐Chair (Milan), Ahmed Toosy, MD, PhD (London).

## Supporting information


**Figure S1:** Histograms showing the age distribution of healthy subjects forming the brain age modeling cohort for the training (top), validation (middle), and testing (bottom) subsets. The male: female ratios of the training/validation/testing subsets were 1.36/1.19/1.18.
**Figure S2:** Outline of the DenseNet architecture design. The Dense block is shown on the left and the overall network is on the right. Conv = 3D convolutional layer. Concat = concatenation, FC = fully connected neural network layer(s) (Huang et al. 2016).
**Figure S3:** 3D Inception‐ResNet‐V2 architecture design. (A) shows the overall architecture of the convolutional neural network using different blocks, with reduction blocks used to reduce matrix size and aggregate features. The matrix is reduced in size until it measures 4 × 5 × 4 where an average pool is taken and entered into a fully connected neural network which outputs the age estimate. (B) shows the initial stem architecture. (C) shows the different CNN analysis structures repeated in A. The convolutional blocks (and stem) were altered to allow for analysis of a 3D image, adding an additional layer for the extra dimension axis. FC = fully connected NN layer(s). Conv = 3D convolutional layer, concat = concatenation (Szegedy et al., 2017).
**Figure S4:** Distributions of absolute errors in age prediction for the FLAIR x5 ensemble of the Inception‐ResNet‐V2, the x5 ensemble T1w model, and the combined FLAIR x5 + T1w x5 ensemble model. There was no significant difference between models using a T‐test (FLAIR vs. T1w *p* = 0.90, FLAIR vs. T1w&FLAIR *p* = 0.30). The standard deviation of the absolute error was calculated with bootstrapping and used to perform a T‐test, showing no significant difference across modalities.
**Figure S5:** (A) FLAIR of a subject with white matter lesions, (B) Same MRI as A with lesions filled, (C) Automatically generated white matter lesion masked used to fill lesions.
**Figure S6:** Group average saliency maps for models obtained from skull‐stripped images. Saliency maps for FLAIR‐based (top) and T1w‐based (bottom) trained on skull‐stripped images with InceptionNet, normalised to the MNI space, smoothed, and averaged across subjects. Unthresholded maps are superimposed on the MNI brain template (scale displayed on the right).
**Table S1:** Performance in the test set of Inception‐ResNet‐V2 models trained and evaluated on images preprocessed according to different pipelines. Standard pre‐processing consisted of affine registration to MNI space, N4 bias correction, and no skull stripping.
**Table S2:** Table comparing accuracy measures of different combinations of FLAIR and T1w Inception‐ResNet‐V2 models. To reduce the variance in the predictions, the same network was trained ten times: the best model was chosen based on the lowest validation set MAE, and the five best‐performing models were combined in an ensemble by averaging their predictions. The *p* values are reported for the difference between ensemble and single best models. The FLAIR x5 + T1w x5 ensemble model was significantly more accurate than the single best FLAIR (*p* = 0.03) and single best T1w (*p* = 0.05) models. There were no significant differences for the FLAIR x5 + T1w x5 ensemble vs. the FLAIR x5 or T1w ensembles (*p* > 0.30).
**Table S3:** FLAIR Inception‐ResNet‐v2 brain age Saliency of atlas areas with > 100 highly salient voxels.
**Table S4:** T1w Inception‐ResNet‐v2 brain age model saliency of atlas areas with > 100 highly salient voxels.
**Table S5:**. Comparison of the FLAIR and T1w Inception Net models evaluated on MRI scans with and without lesion filling. Standard deviations were calculated with 1000 sample bootstrapping of the mean absolute error and *p* values were obtained with T‐tests.
**Table S6:** Correlation matrix of FLAIR‐ and T1w‐based brain‐PAD values with established MRI‐derived measures of MS severity, namely total lesion volume (TLV) and brain parenchymal fraction (BPF), obtained in the MS cohort. Pearson correlations were adjusted for age and gender. ***p < 0.001.

## Data Availability

The data that support the findings of this study are available on request from the corresponding author. The data are not publicly available due to privacy or ethical restrictions. Models developed in the study and all relevant code are available at https://github.com/jordan‐colman/FLAIR‐Brain‐Age.
