# Supplementary material for: Brain Age Estimation on T2‐FLAIR Scans for Application to Multiple Sclerosis
Source: Hum Brain Mapp. 2026 Mar 19;47(5):e70425. doi: 10.1002/hbm.70425 (PMC13081688; doi:10.1002/hbm.70425)
Supplement: Supplementary file 1 — Figure S1: Histograms showing the age distribution of healthy subjects forming the brain age modeling cohort for the training (top), validation (middle), and testing (bottom) subsets. The male: female ratios of the training/validation/testing subsets were 1.36/1.19/1.18. Figure S2: Outline of the DenseNet architecture design. The Dense block is shown on the left and the overall network is on the right. Conv = 3D convolutional layer. Concat = concatenation, FC = fully connected neural network layer(s) (Huang et al. 2016). Figure S3: 3D Inception‐ResNet‐V2 architecture design. (A) shows the overall architecture of the convolutional neural network using different blocks, with reduction blocks used to reduce matrix size and aggregate features. The matrix is reduced in size until it measures 4 × 5 × 4 where an average pool is taken and entered into a fully connected neural network which outputs the age estimate. (B) shows the initial stem architecture. (C) shows the different CNN analysis structures repeated in A. The convolutional blocks (and stem) were altered to allow for analysis of a 3D image, adding an additional layer for the extra dimension axis. FC = fully connected NN layer(s). Conv = 3D convolutional layer, concat = concatenation (Szegedy et al., 2017). Figure S4: Distributions of absolute errors in age prediction for the FLAIR x5 ensemble of the Inception‐ResNet‐V2, the x5 ensemble T1w model, and the combined FLAIR x5 + T1w x5 ensemble model. There was no significant difference between models using a T‐test (FLAIR vs. T1w p = 0.90, FLAIR vs. T1w&FLAIR p = 0.30). The standard deviation of the absolute error was calculated with bootstrapping and used to perform a T‐test, showing no significant difference across modalities. Figure S5: (A) FLAIR of a subject with white matter lesions, (B) Same MRI as A with lesions filled, (C) Automatically generated white matter lesion masked used to fill lesions. Figure S6: Group average saliency maps for models obtained from s [file HBM-47-e70425-s001.docx]

**Brain Age Estimation on T2-FLAIR Scans**

**for Application to Multiple Sclerosis**

**Supplementary Methods**

# Brain Age Modelling

## Pre-processing

MR images were pre-processed by correcting for intensity inhomogeneity using N4 correction with the ‘niftkBiasFieldCorrection’ tool in NifTK[Clarkson et al., 2014], and then aligned to 1mm isotropic MNI space to ensure all MRIs were at the same resolution and orientation. Alignment was performed using NifTK with affine registration of the T1w MRI to the MNI152 T1w 1mm image using the ‘reg_aladdin’ tool.[Clarkson et al., 2014] FLAIR scans were initially aligned to the corresponding T1w image, the resulting matrix was then multiplied with the T1w to MNI space matrix, and the original FLAIR image was transformed by the resulting matrix in a single step to improve registration accuracy and reduce interpolation error. Skull stripping, if applied, was performed by multiplying transformed images by a dilated brain mask of the MNI152 image (included with FSL) using FSL’s ‘fslmaths’[Jenkinson et al., 2012]. This was done due to potential errors that may occur when performing skull stripping on an individual level, which may remove brain tissue and is not feasible for visual quality checks due to the large amount of data.

## Data augmentation

Prior to data augmentation, data is normalised by subtracting the mean and dividing by the standard deviation, and then scaled so image values are between 0 and 1. Data augmentation was performed using the Monai Transforms library (<https://docs.monai.io/en/latest/transforms.html>). Multiple random transformations were used:

- Randomly scaling the whole image by factor randomly selected from 0.8 to 1.2 using ‘RandScaleIntensity’ function with a probability of 0.25;
- Randomly shifting the whole image by factor randomly selected from -0.2 to 0.2 using ‘RandShiftIntensity’ function with a probability of 0.25;
- Randomly flipping the image in the Left to Right direction with a probability of 0.50;
- Randomly shifting points on the histogram of the image using ‘RandHistogramShift’ with a probability of 0.1;
- Randomly adjusting the contrast of the image (‘RandAdjustContrast’) where the image is updated as image = ((image – min_value) /intensity_rage )^gamma * intensity_range + min_value, where gamma is a random value selected between 0.5 and 4.5 and there is a 0.1 probability of the contrast being randomly altered;
- Randomly performing a gaussian smooth (‘RandGaussianSmooth’) of the image with a probability of 0.1 with a sigma randomly selected from the range 0.25 to 1.5 spatial dimensions (voxel size);
- Randomly adding gaussian noise (‘RandGaussianNoise’) with a probability of 0.1, mean = 0.0 and standard deviation = 0.1;
- Randomly Shifting the image by 0-3 voxels in each direction with equal probability, performed by randomly cropping a region smaller than 3 voxels along each dimension from the original image using the ‘RandomSpatialCrop’ function (Cropped at the centre of the image with ‘CentreSpatialCrop’ when evaluating model performance).

## CNN Architectures

### SFCN

The SFCN network was taken from the corresponding Github repository (<https://github.com/ha-ha-ha-han/UKBiobank_deep_pretrain>) and the architecture outlined in the corresponding publication^3^. Briefly, it consists of 5 3D convolutional layers with 3D kernels sized 3x3x3 which are followed by Batch normalisation, MaxPool 2x2x2 (which downsamples the image by a factor of 2 in each spatial dimension), and Relu operations. The model outputs a 40-class vector with each class representing an age value over the potential age range 15-95 with gaps of 2 years between each class. The whole vector can be thought of as a probability density function of predicted age and the most probable age calculated by $\sum_{c}^{40} X_{c}*{Age}_{c}$X_c_ is the probability of the age at class c and Age_c_ is the Age represented by class c.[Peng et al., 2021]

### DenseNet

The DenseNet is a much more complex or deep network than the SFCN, with many more trainable parameters[Huang et al., 2016]. It connects the output of every layer to the input of every other layer, this gives each convolutional layer access to the ‘knowledge’ of all previous layers. The DenseNet used had 169 convolutional layers and was available as a pre-built network in the Monai Library (<https://docs.monai.io/en/latest/_modules/monai/networks/nets/densenet.html>). The overview of the network design can be seen in Supplementary Figure 2. Unlike the SFCN architecture, the network ends with a series of fully connected layers and outputs a single floating-point output with linear regression applied (i.e., predicted age).

### Inception-ResNet V2

The Inception-ResNet-V2 network, also referred to as InceptionNet in this work, consists of various ‘Modules’ (A, B, and C), meant to increase the ‘field of view’ of the kernels in the layer without increasing computational complexity[Szegedy et al., 2017]. The original 2D Inception-Resnet V2 was altered to run in 3 spatial dimensions using the pytorch library[Paszke, Adam and Gross, Sam and Massa, Francisco and Lerer, Adam and Bradbury, James and Chanan, Gregory and Killeen, Trevor and Lin, Zeming and Gimelshein, Natalia and Antiga, Luca and Desmaison, Alban and Kopf, Andreas and Yang, Edward and DeVito, Zacha, 2019] (Supplementary Figure 3): module A performs two subsequent 3x3x3 sized kernel convolutions; in module B, three subsequent 7x1x1, 1x7x1, and 1x1x7 convolutions are used to cover a 7x7x7 sized field of view while being less computationally complex. The Inception Net additionally makes use of identity mapping/residual connections and multiplies the blocks’ output by a factor to scale it to a smaller size compared to the skip connection (in this case, a factor of 0.2 was used).

## Experimental Setup

All experiments were performed in python using Pytorch[Paszke, Adam and Gross, Sam and Massa, Francisco and Lerer, Adam and Bradbury, James and Chanan, Gregory and Killeen, Trevor and Lin, Zeming and Gimelshein, Natalia and Antiga, Luca and Desmaison, Alban and Kopf, Andreas and Yang, Edward and DeVito, Zacha, 2019]. The Monai library was used to read MR images and perform data standardisation and augmentation as described above[MONAI, 2020]. All experiments were run on the UCL high-performance cluster on a GPU with a RAM of 12Gb (<https://hpc.cs.ucl.ac.uk/>)[UCL CS, 2021].

### SFCN

The SFCN network was trained using parameters described in the paper describing the network.[Peng et al., 2021] The stochastic gradient descent (SGD) optimiser was used with an initial learning rate of 0.01, L2 weight decay coefficient of 0.001, and scheduled weight decay multiply the learning rate by 0.3 every 3 epochs, using KL-divergence loss.[Peng et al., 2021] The input images were kept at a 1mm isotropic resolution and cropped to a size of 160,192,160. The age label was changed to a Gaussian estimate centred around the true age on a 40-long vector with each point representing a 2-year interval from 15 to 95. The training was run for 130 epochs with a batch size of 3 due to memory limitations. The model was trained with either random initial weights or with pre-trained weighs from the network being trained on the UK Biobank data (model code and pre-trained weights available at: <https://github.com/ha-ha-ha-han/UKBiobank_deep_pretrain>).

### DenseNet and InceptionNet

Initially, the data was interpolated in MNI space to a smaller resolution (size 121,145,121, corresponding to 1.45mm isotropic resolution and then centre-cropped to a size of 118,142,121) to reduce dimensionality and increase the batch size. The Networks were optimised using the adaptive moment estimation (ADAM) optimizer, with an initial learning rate of 1e-4 and a linear decay as described in *Chen et al.*,[Chen et al., 2016] with subsequent learning rates equalling:

$$\begin{aligned} Initial\_learing\_rate* \left( 1-\left( \frac{epoch}{Max_{epoch}} \right) \right)^{0.9}\# \end{aligned}$$

Where Initial_learning_rate = 1e-4, epoch is the current epoch number, and max_epoch is 200. Additional L2 regularisation was applied at a rate of 1e-5. The batch size was 10, the models were trained for 200 epochs and the weights of the epoch with the lowest MAE on the validation set were saved and used for evaluation on the test set.

## Age bias correction

The age bias of the models (i.e. underestimation of the age of older subjects and vice versa) was quantified by calculating the Pearson’s correlation coefficient of the chronological age and brain-PAD in the validation set. To correct for age bias, a linear fit between predicted and chronological age was estimated in the validation set

$$\begin{aligned} predicted\_brain\_age= A*Chronological\_age- B\# \end{aligned}$$

and its coefficients were used to correct external predictions

$$\begin{aligned} corrected\_predicted\_brain\_age =\frac{predicted\_brain\_age - B}{A} \# \end{aligned}$$

This procedure reduces age bias for a small decrease in model accuracy.[Peng et al., 2021]

## Saliency mapping

Saliency mapping was performed using the SmoothGrad method[Smilkov et al., 2017] as described in *Levakov et al.*[Levakov et al., 2020]. The calculated gradient maps were standardised so that the image mean = 0 and SD = 1. The maps were then spatially normalised by nonlinear registration to the MNI space at a 1mm isotropic resolution, and a Gaussian smoothing was applied with a 4mm full width at half maximum.[Levakov et al., 2020] This was performed for all subjects of the test set, and values were averaged across individuals. Saliency maps were obtained for all FLAIR and T1w Inception-ResNet-v2 ensemble models and the average ensemble saliency values were taken. As described in *Levakov et al.*[Levakov et al., 2020], the group average saliency maps were threshold to the highest 1% of voxels to show areas that are the highly salient for predicting brain age. Atlases were then used to measure the number of highly salient voxels in each brain region and to get the mean saliency of the regions. Specifically, the Desikan-Killiany-Tourville (DKT) atlas was used for the grey matter structures and ventricles, and the ICBM DTI-81 for white matter structures[Levakov et al., 2020].

# References

Chen L-C, Papandreou G, Kokkinos I, Murphy K, Yuille AL (2016): DeepLab: Semantic Image Segmentation with Deep Convolutional Nets, Atrous Convolution, and Fully Connected CRFs. IEEE Transactions on Pattern Analysis and Machine Intelligence 40:834–848.

Clarkson MJ, Zombori G, Thompson S, Totz J, Song Y, Espak M, Johnsen S, Hawkes D, Ourselin S (2014): The NifTK software platform for image-guided interventions: platform overview and NiftyLink messaging. International Journal of Computer Assisted Radiology and Surgery 2014 10:3 10:301–316.

Huang G, Liu Z, van der Maaten L, Weinberger KQ (2016): Densely Connected Convolutional Networks. Proceedings - 30th IEEE Conference on Computer Vision and Pattern Recognition, CVPR 2017 2017-January:2261–2269.

Jenkinson M, Beckmann CF, Behrens TEJ, Woolrich MW, Smith SM (2012): FSL. NeuroImage 62:782–90.

Levakov G, Rosenthal G, Shelef I, Raviv TR, Avidan G (2020): From a deep learning model back to the brain—Identifying regional predictors and their relation to aging. Human Brain Mapping 41:3235–3252.

MONAI P (2020): The MONAI Consortium. Zenodo.

Paszke, Adam and Gross, Sam and Massa, Francisco and Lerer, Adam and Bradbury, James and Chanan, Gregory and Killeen, Trevor and Lin, Zeming and Gimelshein, Natalia and Antiga, Luca and Desmaison, Alban and Kopf, Andreas and Yang, Edward and DeVito, Zacha S (2019): PyTorch: An Imperative Style, High-Performance Deep Learning Library. In: . Advances in Neural Information Processing Systems 32. Curran Associates, Inc. pp 8024–8035.

Peng H, Gong W, Beckmann CF, Vedaldi A, Smith SM (2021): Accurate brain age prediction with lightweight deep neural networks. Medical image analysis 68:101871.

Smilkov D, Thorat N, Kim B, Viégas F, Wattenberg M (2017): SmoothGrad: removing noise by adding noise.

Szegedy C, Ioffe S, Vanhoucke V, Alemi AA (2017): Inception-v4, Inception-ResNet and the Impact of Residual Connections on Learning. Thirty-First AAAI Conference on Artificial Intelligence.

UCL CS UCSRSG (2021): UCL Computer Science High Performance Computing Service. https://hpc.cs.ucl.ac.uk/.

# Supplementary Figures


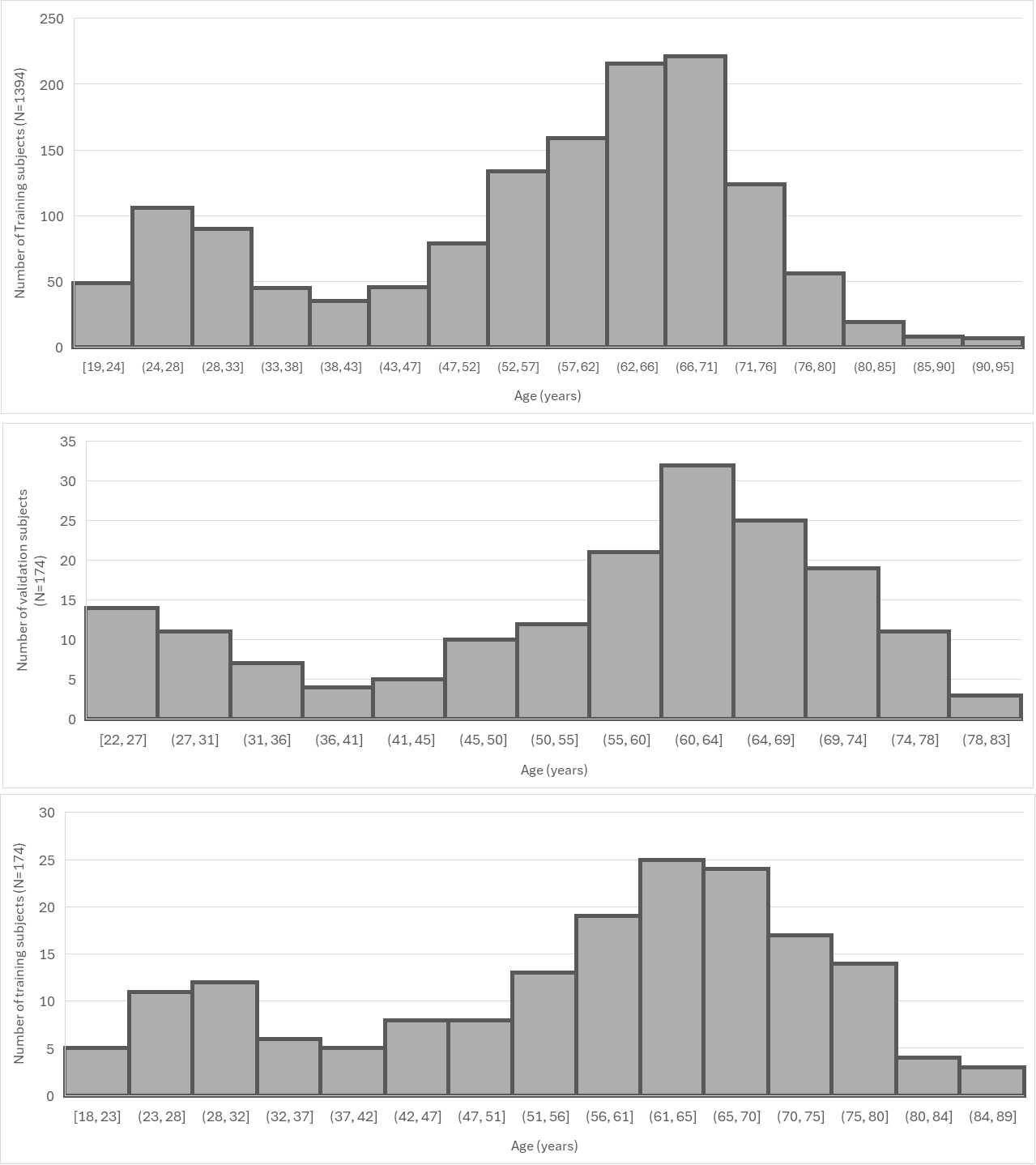


**Supplementary Figure 1.** Histograms showing the age distribution of healthy subjects forming the brain age modelling cohort for the training (top), validation (middle), and testing (bottom) subsets. The male:female ratios of the training/validation/testing subsets were 1.36/1.19/1.18.


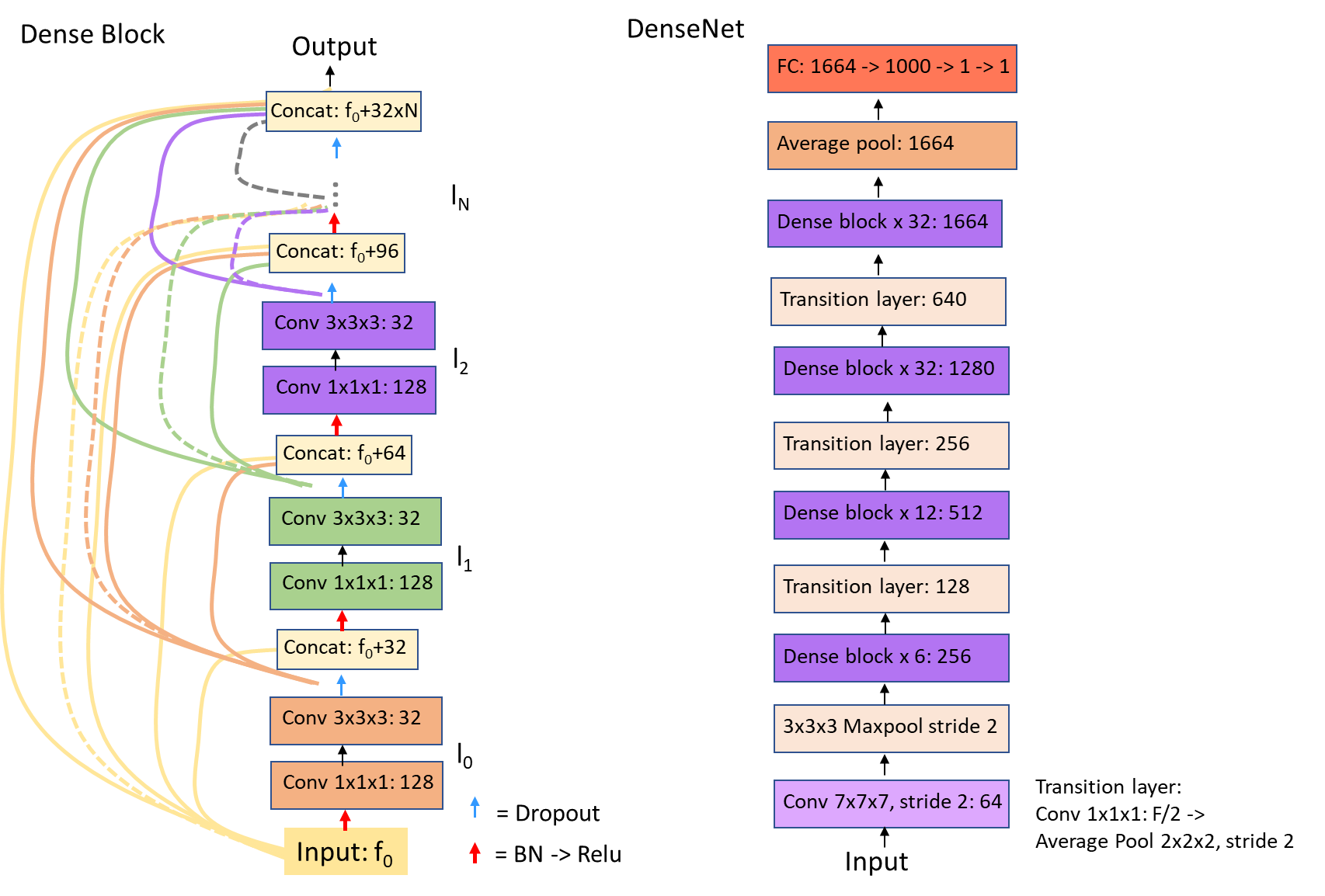


**Supplementary Figure 2. Outline of the DenseNet architecture design.** The Dense block is shown on the left and the overall network is on the right. Conv = 3D convolutional layer. Concat = concatenation, FC = fully connected neural network layer(s).[Huang et al., 2016]


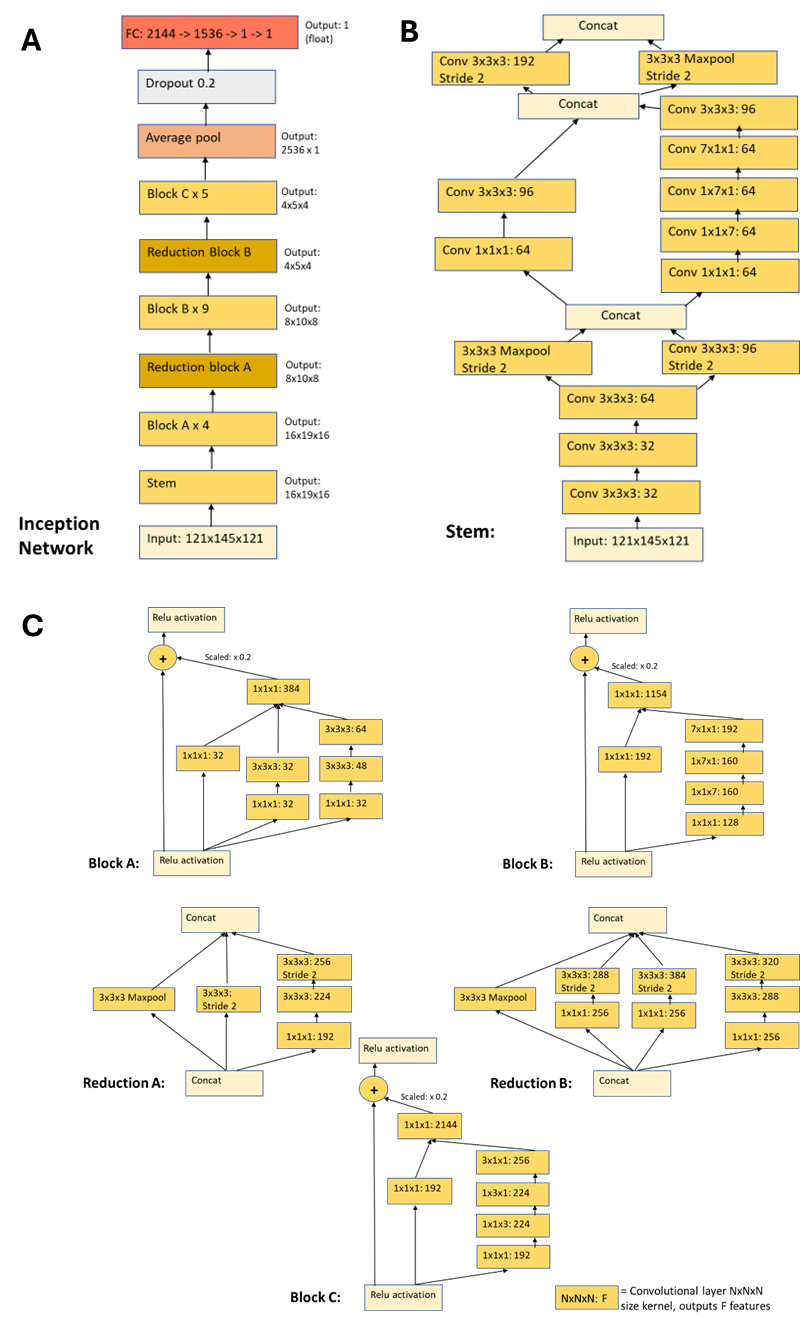


**Supplementary Figure 3. 3D Inception-ResNet-V2 architecture design.** A: shows the overall architecture of the convolutional neural network using different blocks, with reduction blocks used to reduce matrix size and aggregate features. The matrix is reduced in size until it measures 4x5x4 where an average pool is taken and entered into a fully connected neural network which outputs the age estimate. B: shows the initial stem architecture. C: shows the different CNN analysis structures repeated in A. The convolutional blocks (and stem) were altered to allow for analysis of a 3D image, adding an additional layer for the extra dimension axis. FC = fully connected NN layer(s). Conv= 3D convolutional layer, concat= concatenation[Szegedy et al., 2017].


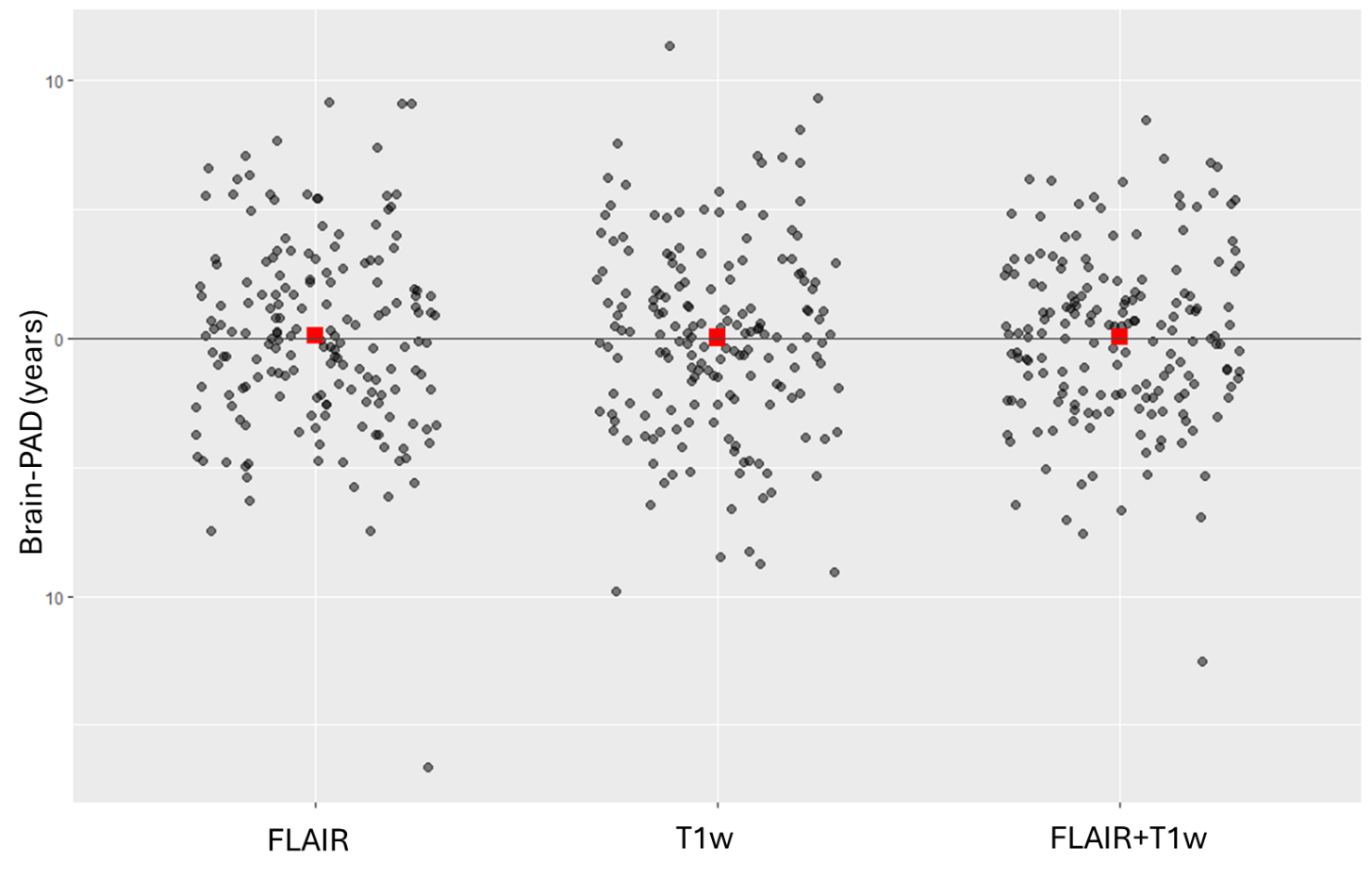


**Supplementary Figure 4.** Distributions of absolute errors in age prediction for the FLAIR x5 ensemble of the Inception-ResNet-V2, the x5 ensemble T1w model, and the combined FLAIR x5 + T1w x5 ensemble model. There was no significant difference between models using a T-test (FLAIR vs T1w p=0.90, FLAIR vs T1w&FLAIR p=0.30). The standard deviation of the absolute error was calculated with bootstrapping and used to perform a T-test, showing no significant difference across modalities.


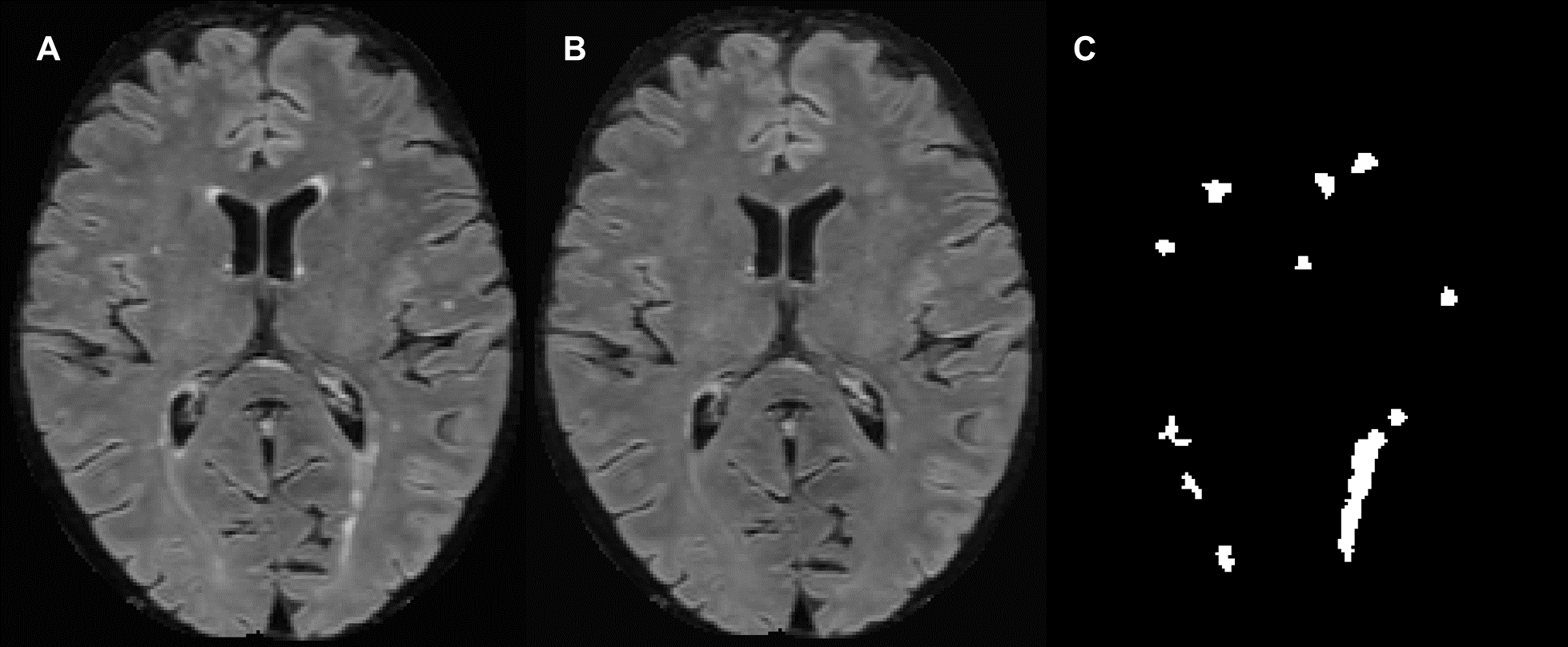


**Supplementary Figure 5.** A: FLAIR of a subject with white matter lesions, B: Same MRI as A with lesions filled, C: Automatically generated white matter lesion masked used to fill lesions.


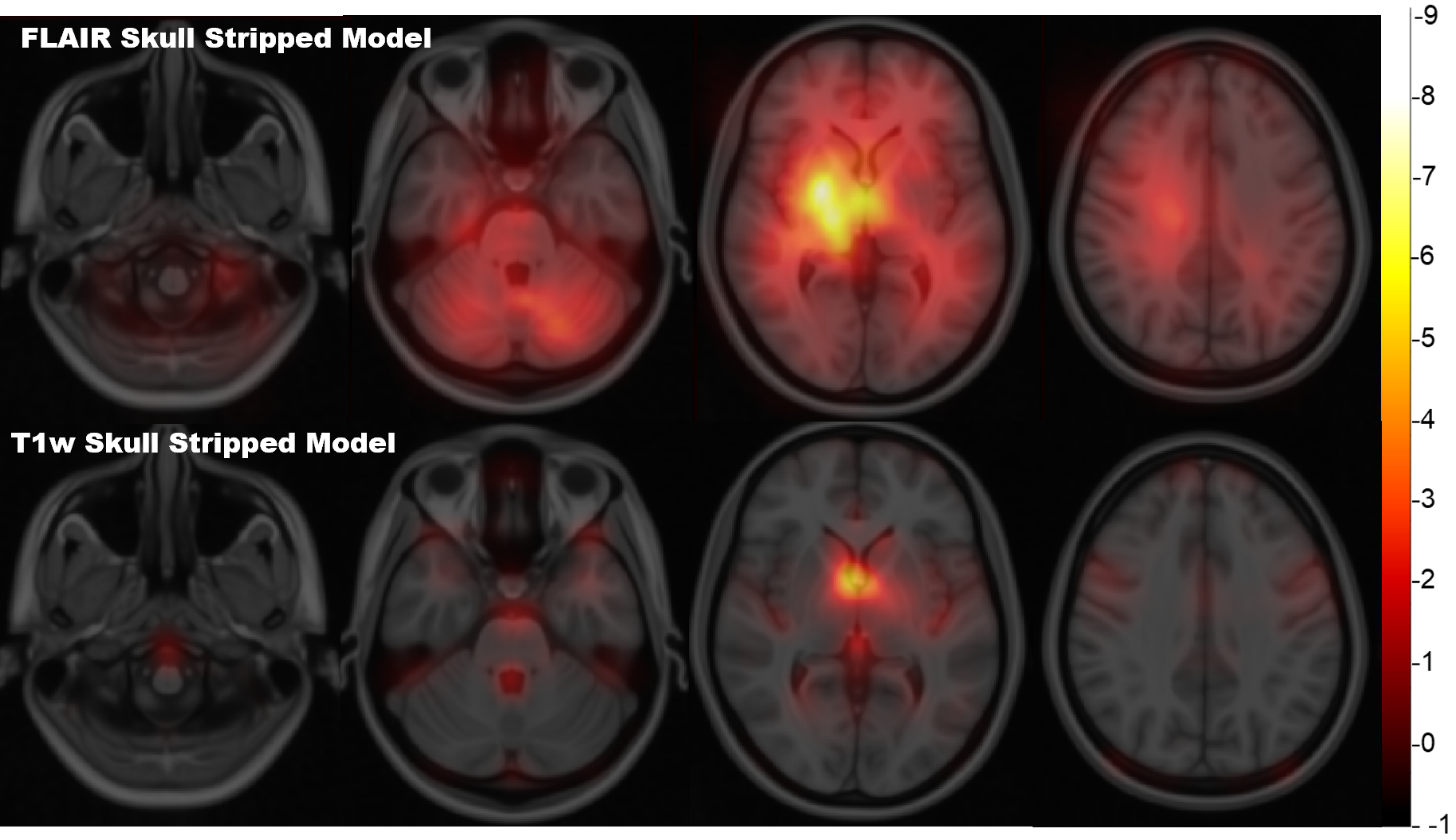


**Supplementary Figure 6. *Group average saliency maps for models obtained from skull-stripped images.*** *Saliency maps for FLAIR-based (top) and T1w-based (bottom) trained on skull-stripped images with InceptionNet, normalised to the MNI space, smoothed, and averaged across subjects. Unthresholded maps are superimposed on the MNI brain template (scale displayed on the right).*

# Supplementary Tables

|  | **FLAIR** | | | | **T1w** | | | |
| --- | --- | --- | --- | --- | --- | --- | --- | --- |
| **Pre-processing** | **MAE** | **R** | **R^2^** | **p-value** | **MAE** | **R** | **R^2^** | **p-value** |
| Standard | 3.31 +/- 2.44 | 0.972 | 0.944 | - | 3.34 +/- 2.47 | 0.971 | 0.942 | - |
| Rigid registration | 3.54 +/- 2.83 | 0.964 | 0.930 | 0.89 | 3.32 +/- 2.77 | 0.969 | 0.939 | 0.94 |
| Skull stripping | 3.44 +/- 2.65 | 0.968 | 0.937 | 0.63 | 4.44 +/- 3.31 | 0.947 | 0.896 | 0.0005 |

**Supplementary Table 1.** Performance in the test set of Inception-ResNet-V2 models trained and evaluated on images preprocessed according to different pipelines. Standard pre-processing consisted of affine registration to MNI space, N4 bias correction, and no skull stripping.

|  | **MAE (years)** | **R** | **R^2^** | **p-value** |
| --- | --- | --- | --- | --- |
| Best FLAIR model | 3.10 +/- 2.42 | 0.974 | 0.948 | - |
| FLAIR x5 ensemble | 2.81 +/- 2.34 | 0.977 | 0.955 | 0.26 |
| Best T1w model | 3.04 +/- 2.46 | 0.975 | 0.951 | - |
| T1w x5 ensemble | 2.84 +/- 2.33 | 0.977 | 0.955 | 0.44 |
| Best FLAIR + T1w model | 2.83 +/- 2.10 | 0.979 | 0.958 | - |
| FLAIR x5 + T1w x5 ensemble | 2.57 +/- 2.00 | 0.982 | 0.965 | 0.22 |

***Supplementary Table 2.*** *Table comparing accuracy measures of different combinations of FLAIR and T1w Inception-ResNet-V2 models. To reduce the variance in the predictions, the* *same network was trained ten times: the best model was chosen based on the lowest validation set MAE, and the five best-performing models were combined in an ensemble by averaging their predictions.* *P values are reported for the difference between ensemble and single best models. The FLAIR x5 + T1w x5 ensemble model was significantly more accurate than the single best FLAIR (p = 0.03) and single best T1w (p = 0.05) models. There were no significant differences for the FLAIR x5 + T1w x5 ensemble vs the FLAIR x5 or T1w ensembles (p > 0.30).*

| **FLAIR CNN brain age model Salient anatomical regions** | **Voxels over Saliency threshold** | **Voxels over Saliency threshold as proportion (%)** | **Mean Saliency Map Value** | **SD of Saliency Map Value** |
| --- | --- | --- | --- | --- |
| Right thalamus proper | 7424 | 86.44 | 5.50 | 1.39 |
| Right ventral diencephalon | 3382 | 66.63 | 4.44 | 1.02 |
| Posterior limb of internal capsule right | 2820 | 75.16 | 5.51 | 1.77 |
| Right putamen | 1956 | 28.46 | 3.54 | 1.06 |
| Right pallidum | 1797 | 99.61 | 5.73 | 0.88 |
| 3rd ventricle | 1379 | 71.05 | 4.43 | 0.78 |
| External capsule right | 1021 | 27.97 | 3.36 | 1.05 |
| Right lateral ventricle | 998 | 5.06 | 2.14 | 0.91 |
| Cerebral peduncle right | 938 | 41.18 | 3.85 | 1.28 |
| Left thalamus proper | 609 | 7.07 | 2.91 | 0.74 |
| Anterior limb of internal capsule right | 547 | 18.12 | 3.08 | 0.80 |
| Retrolenticular part of internal capsule right | 471 | 19.08 | 2.66 | 1.47 |
| Fornix | 313 | 47.50 | 3.71 | 0.81 |
| Brain stem | 262 | 0.98 | 1.87 | 0.73 |
| Left lateral ventricle | 181 | 0.84 | 1.56 | 0.73 |
| Inferior fronto-occipital fasciculus right | 121 | 6.25 | 2.68 | 0.69 |

**Supplementary Table 3.** FLAIR Inception-ResNet-v2 brain age Saliency of atlas areas with >100 highly salient voxels.

| **T1w CNN brain age model Salient anatomical regions** | **Voxels over Saliency threshold** | **Voxels over Saliency threshold as proportion (%)** | **Mean Saliency Map Value** | **SD of Saliency Map Value** |
| --- | --- | --- | --- | --- |
| Right ventral diencephalon | 3138 | 61.82 | 4.01 | 1.27 |
| Right thalamus proper | 2355 | 27.42 | 2.95 | 0.77 |
| Right lateral ventricle | 1219 | 6.19 | 1.43 | 0.96 |
| 3rd ventricle | 1036 | 53.37 | 3.51 | 1.02 |
| Cerebral peduncle right | 845 | 37.09 | 3.31 | 1.29 |
| Posterior limb of internal capsule right | 833 | 22.20 | 3.00 | 0.71 |
| Anterior limb of internal capsule right | 479 | 15.87 | 2.22 | 1.06 |
| Right pallidum | 395 | 21.90 | 2.94 | 0.68 |
| Right amygdala | 221 | 9.915 | 2.44 | 0.62 |
| Right caudate | 198 | 5.140 | 1.92 | 0.82 |
| Right accumbens area | 141 | 18.63 | 2.97 | 0.45 |
| Left ventral diencephalon | 139 | 2.70 | 2.37 | 0.56 |
| Brain stem | 110 | 0.41 | 1.74 | 0.53 |

**Supplementary Table 4.** T1w Inception-ResNet-v2 brain age model saliency of atlas areas with >100 highly salient voxels.

|  | Raw Images MAE (± SD) | Lesion Filled Images MAE (± SD) | p-value |
| --- | --- | --- | --- |
| FLAIR | 3.31 ± 2.44 | 3.35 ± 2.53 | 0.881 |
| T1 | 3.35 ± 2.41 | 3.36 ± 2.44 | 0.982 |

**Supplementary Table 5.** Comparison of the FLAIR and T1w Inception Net models evaluated on MRI scans with and without lesion filling. Standard deviations were calculated with 1000 sample bootstrapping of the mean absolute error and p values were obtained with T-tests.

|  | FLAIR brain-PAD | T1w brain-PAD | TLV | BPF |
| --- | --- | --- | --- | --- |
| FLAIR brain-PAD | ~ | 0.748^***^ | 0.610^***^ | -0.600^***^ |
| T1w brain-PAD | 0.748^***^ | ~ | 0.475^***^ | -0.493^***^ |
| TLV | 0.610^***^ | 0.475^***^ | ~ | -0.529^***^ |
| BPF | -0.600^***^ | -0.493^***^ | -0.529^***^ | ~ |

**Supplementary Table 6.** Correlation matrix of FLAIR- and T1w-based brain-PAD values with established MRI-derived measures of MS severity, namely total lesion volume (TLV) and brain parenchymal fraction (BPF), obtained in the MS cohort. Pearson correlations were adjusted for age and gender. *** p < 0.001
